# Barriers and facilitators of mental health help-seeking behaviours among school-going adolescents in Sub-Saharan Africa: A qualitative evidence synthesis

**DOI:** 10.1017/gmh.2026.10250

**Published:** 2026-06-09

**Authors:** Adrian Ivan Kakinda, Tim J. Croudace, Rachel A. Plouffe, Kennedy Amone-P’Olak

**Affiliations:** 1 Division of Psychology, School of Humanities, Social Sciences and Law, University of Dundee, UK; 2 Division of Health Sciences, Faculty of Health, University of Dundee, UK; 3 Department of Psychology, Faculty of Social Sciences, Kyambogo University, Uganda

**Keywords:** school-going adolescents, mental health, help-seeking behaviours, Sub-Saharan Africa, barriers, facilitators, qualitative evidence synthesis

## Abstract

The prevalence of mental health problems (MHPs) among school-going adolescents in Sub-Saharan Africa (SSA) remains high. However, help-seeking behaviours are disproportionately low in this population. This qualitative evidence synthesis (QES) aimed to identify and integrate existing qualitative findings on the barriers and facilitators of formal and informal mental health (MH) help-seeking behaviours in this population. The objective was to generate insights from relevant studies and settings to inform the development of effective interventions for culturally grounded public mental health initiatives in schools in SSA. We conducted a systematic search across six databases (PubMed, CINAHL, Scopus, PsycINFO, ERIC and Google Scholar) for qualitative studies published until September 2025. Eligible studies were appraised using the Critical Appraisal Skills Programme (CASP) checklist. Thematic synthesis was employed to analyse and interpret the findings. Twelve studies met the inclusion criteria. Six key barriers were identified: (1) perceived stigma, (2) gender norms as a barrier to help-seeking, (3) poor mental health knowledge, misconceptions and awareness gaps, (4) privacy, trust and confidentiality concerns with MH professionals, (5) lack of accessibility and availability of MH services and (6) family and parental attitudes, peer influence and alternative support. Facilitators included (1) MH education and literacy enhancement, (2) supportive school environment or climate, (3) improved professional services, (4) family, community involvement and peer support and (5) improved service accessibility and affordability. This synthesis highlights the significant structural and sociocultural determinants of help-seeking behaviour in school-going adolescents in SSA. The scarcity of qualitative studies in this area underscores a critical gap in the existing literature. Further context-sensitive qualitative research is urgently needed to gain insights into adolescents’ lived experiences with MHPs and to guide responsive school-based MH interventions.

## Graphical abstract

**Figure:**
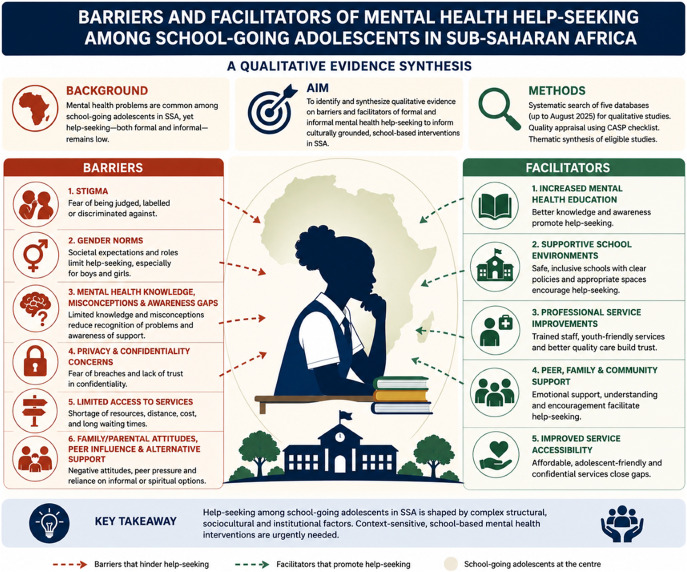

## Impact Statements

This QES consolidates evidence on barriers and facilitators of school-based mental health help-seeking among adolescents in SSA, where a high burden of MHPs has been reported. Although schools are positioned as cost-effective entry points for support, the findings indicate that service presence alone does not translate into utilisation. Adolescents interpret help-seeking through the relational, cultural and institutional conditions within which services are embedded. The synthesis identifies the interconnected structural and individual factors that shape poor help-seeking. Adolescents’ concerns centre on mistrust, weak rapport and prior negative experiences with providers. Social and contextual influences included perceived stigma, gender norms, low mental health literacy, limited accessibility to care, parental/familial authority and expectations, peer influence and reliance on informal or spiritual support systems. Across studies, adolescents assess disclosure risks, including fear of judgement, confidentiality breaches and social consequences, before engaging with formal services. Help-seeking is socially constructed and largely dependent on experienced and perceived relational safety rather than individual unwillingness to seek help. Interventions that prioritise awareness without addressing trust, confidentiality and institutional credibility are unlikely to improve uptake. School systems should operate as context-sensitive platforms that incorporate confidentiality protocols, trust-building strategies, clearly defined roles within school systems and active family and community involvement at each stage of support, aligned with cultural contexts. For policymakers, the QES supports a shift from service provision metrics to standards that assess relational safety, cultural context and accountability within school-based systems. For researchers, this review highlights the limited qualitative evidence and underrepresentation of adolescents’ perspectives, requiring context-sensitive research that centres lived experiences to inform intervention design.

## Introduction

Mental health problems (MHPs), such as depression and anxiety, account for a sizeable proportion of the global disease burden among adolescents, with up to 14% of 10- to 19-year-olds affected worldwide (Jessiman et al., 2022; Amone-P’Olak et al., 2023; Akın and Sarrar, 2024; Cosma et al., 2025). Despite this high prevalence, many adolescents do not access either professional help or informal support. This is particularly pronounced in Sub-Saharan Africa (SSA), where health systems are under-resourced, MH services are sparse and stigma remains widespread (Babatunde et al., 2021; Al Omari et al., 2022; Barrow and Thomas, 2022).

While adolescence is a critical period for the emergence of MHPs, it also represents an important window for early intervention and the commencement of long-term MH promotion (Addy et al., 2021; Al Omari et al., 2022; Birrell et al., 2025). Most school-going adolescents in SSA cannot access formal MH care (Addy et al., 2021; Khombo et al., 2023). This disconnect has been highlighted (Al Omari et al., 2022; O’Neill et al., 2023), prompting concerns among researchers and practitioners regarding untreated MHPs (Mutahi et al., 2022; Hayes et al., 2024; Nguyen et al., 2025). Adolescents who do not receive prompt support for emerging MHPs are more likely to face academic disruption, social exclusion, early parenthood, exposure to violence and enduring psychiatric conditions in adulthood (Abdulsalam et al., 2023; Al-Shannaq  and Aldalaykeh, 2023). The implications of low help-seeking therefore extend beyond immediate emotional or behavioural problems to include long-term psychological disorders, impaired social and academic functioning and increased public health burdens (Seedaket et al., 2020; Lu et al., 2021; Radez et al., 2021).

Help-seeking for MHPs refers to the adaptive coping process through which individuals recognise the need for support and engage with formal or informal sources of help (Rickwood et al., 2005). Barriers to help-seeking processes are multifaceted, including intrapersonal factors such as self-stigma and low mental health literacy (MHL), including limited knowledge, misconceptions and awareness of available support (Aguirre Velasco et al., 2020; Barrow and Thomas, 2022), interpersonal constraints such as poor social support or negative family attitudes and structural barriers such as cost, distance and lack of accessible services (Gulliver et al., 2010; Eigenhuis et al., 2021). In contrast, facilitators of help-seeking may include the presence of trusted adults, integration of MHL programmes into the school curriculum, prior positive experiences with help-seeking and culturally appropriate MH messages (Aguirre Velasco et al., 2020; Bach et al., 2023). Although several systematic reviews have explored these themes, most have synthesised data from mixed populations of adolescents or focused exclusively on those with diagnosed conditions rather than considering school-going adolescents as a discrete subgroup (Aguirre Velasco et al., 2020; Radez et al., 2021; Barrow and Thomas, 2022).

Recognising these long-term consequences, the World Mental Health Report (2022) (Freeman, 2022) and the Mental Health Action Plan (2013–2030) (Singh, 2021) have highlighted school-based interventions as potentially cost-effective entry points for introducing support for MH (Singh, 2021; Freeman, 2022; World Health Organization, 2022). In many high-income countries, schools and institutions have become central to early intervention efforts (Duong et al., 2021; Ma et al., 2023; McPhail et al., 2024). A recent systematic review by Hayes et al. (2024) evaluated universal school-based interventions and found tentative yet promising impacts on adolescents’ help-seeking attitudes and intentions, especially through intrapersonal pathways, such as MHL and stigma reduction. However, the review also pointed to numerous limitations, including methodological variations, limited long-term follow-up and a lack of contextual tailoring for most interventions (Hayes et al., 2024).

These limitations are especially pronounced in SSA, where schools often lack key foundational elements, such as embedded MH infrastructure and trained professionals (Jörns-Presentati et al., 2021; Mabrouk et al., 2022). In addition, understanding help-seeking among adolescents in school settings remains limited (Meza et al., 2020; Khombo et al., 2023). These limitations underscore the need for contextually grounded research that explores how help-seeking within school environments is described and discussed in SSA. To date, existing research has predominantly addressed systemic health system limitations, but insufficient attention has been paid to the cultural, individual, institutional or context-specific factors that shape adolescents’ help-seeking behaviours (Meza et al., 2020; Addy et al., 2021; Carlson et al., 2021).

In SSA, where increasing attention is being paid to adolescents’ MH needs, less is known about school-going adolescents’ perspectives on MH experiences and their help-seeking negotiations within the school and sociocultural contexts. Existing research has largely focused on adolescent help-seeking pathways and constraints within health systems, often overlooking school-going adolescents’ perspectives regarding formal and informal support systems within SSA. To address this gap, the present qualitative evidence synthesis (QES) aimed to review and integrate findings from existing studies examining barriers and facilitators associated with MH help-seeking behaviours among school-going adolescents in SSA. In this review, MH help-seeking included formal sources such as school-based counselling and clinical services, as well as informal support systems, including family, peers and community networks. The review was guided by the following question: What barriers and facilitators influence MH help-seeking behaviours among school-going adolescents in SSA?

## Methods

### Review design

Our review of the barriers and facilitators of MH help-seeking behaviours among school-going adolescents in SSA adopted a QES approach. We opted to restrict the review to qualitative studies to provide a rich context for lived experiences, beliefs and attitudes, particularly concerning sensitive topics such as MH help-seeking behaviours (Flemming and Noyes, 2021; Carmona et al., 2022). This enabled the review to explore societal and cultural factors and personal experiences or interactions that shape relevant behaviours in this setting (Long et al., 2020; Flemming and Noyes, 2021).

This review followed the Enhancing Transparency in Reporting the Synthesis of Qualitative Research (ENTREQ) guidelines (Tong et al., 2012; Batten and Brackett, 2022) (see Supplementary Appendix A). The GRADE-CERQual approach (Lewin et al., 2015, 2018) was also used to determine the confidence level of each finding (see Supplementary Appendix D). A PRISMA flow diagram (Page et al., 2021; Haddaway et al., 2022) is included to provide transparency in the reporting of the search and scrutiny processes (Carmona et al., 2022; Akl et al., 2024).

### Framing the question (SPIDER framework)

To specify the review question, we used the SPIDER tool (sample, phenomenon of interest, design, evaluation and research type) (Cooke et al., 2012) to develop a list of search terms (Table 1).

**Table 1.** Application of the SPIDER tool to the review questions Table 1. long description.

Element	Details
Sample (S)	School-going adolescents aged 10–19 years residing in Sub-Saharan Africa
Phenomenon of interest (PI)	Perceived barriers and facilitators influencing formal and informal mental health help-seeking behaviours among school-going adolescents in SSA.
Design (D)	Qualitative studies (focus groups, in-depth interviews, group discussions, paired interviews); mixed-method studies to extract qualitative data.
Evaluation (E)	Perceived barriers, facilitators, views, experiences, attitudes, challenges, hindrances, access and referral processes.
Research type (R)	Peer-reviewed qualitative studies focusing on mental health help-seeking behaviours in SSA.
Keywords	“Help-seeking behaviour” OR “Mental health help-seeking” OR “Utilization of mental health services” OR “Attitude towards seeking help”
	“Access to mental health services” OR “Help-seeking intention” OR “Attitude towards counselling” OR “Attitude towards psychotherapy”
	“Africa/African help-seeking behaviour” OR “Sub-Saharan/Sub-Sahara/Africa south of the Sahara help-seeking behaviour”
	“Africa/African mental health” OR “Sub-Saharan/Sub-Sahara/Africa south of the Sahara help-seeking attitude”
	“Africa/African help-seeking attitude” OR “Africa/African help-seeking intention” OR “Sub-Saharan/Sub-Sahara/Africa south of the Sahara help-seeking intention”
	“Barrier” OR “Challenge” OR “Obstacle” OR “Impediment” OR “Hindrance” OR “Obstruction” OR “Hurdle” OR “Delay” OR “Access” OR “Refer”

### Search strategy and information sources

We searched the PubMed, PsycINFO, Scopus, CINAHL, Google Scholar and ERIC databases. The search combined target variables with the following Medical Subject Headings (MeSH) terms: (1) barriers and facilitators, (2) school-going adolescents, (3) MH, (4) help-seeking and access behaviours, (5) SSA and (6) qualitative studies. The search strategy was developed and adjusted according to the PRISMA guidelines (Aromataris and Riitano, 2014; Bramer et al., 2018; Page et al., 2021). See the Supplementary Material (Supplementary Appendix G) for the search strings for each database and the procedures used. The first search was conducted in March 2024, with updates in July 2024, June 2025 and September 2025. Staged search updates were undertaken to capture newly published qualitative studies prior to final synthesis and submission, ensuring that the review reflects the most current and comprehensive evidence available at the time of reporting.

### Search process and methods

The reference lists of the included articles were screened, and reference management and duplicate removal were conducted using EndNote (Bramer et al., 2017), while study selection was supported using Rayyan (Ouzzani et al., 2016). In addition to database and software-assisted screening procedures (EndNote and Rayyan), manual backward and forward citation searching was conducted to identify relevant qualitative studies that may not have been consistently indexed across databases. Finally, we wrote to the corresponding authors of eligible articles [3] to obtain full-text versions; two full texts were successfully obtained, while one remained unavailable and was thus excluded.

#### Eligibility criteria

Studies were included if they met the following criteria: (1) the sample described was clearly school-going adolescents aged 10–19 years (Singh et al., 2019; World Health Organization, 2018), (2) the population sampled was identified as being at some risk for MHPs and (3) the content examined formal and informal help-seeking behaviours using one or more qualitative methods, such as interviews, focus group discussions, ethnography and so on, to explore subjective experiences, motivations and sociocultural contexts in MH help-seeking. The QES included peer-reviewed studies available in English from 1990 onwards.

Studies outside SSA, those that did not target the specified age group or were not focused on MH help-seeking, along with quantitative studies, were excluded from the synthesis. Articles had to be peer-reviewed, complete and meet qualitative methodology criteria for inclusion. The detailed inclusion and exclusion criteria are presented in Table 2.

**Table 2.** Inclusion and exclusion criteria for the included articles Table 2. long description.

Criteria	Inclusion	Exclusion
Geographic scope	Studies conducted in Sub-Saharan Africa.	Studies conducted outside Sub-Saharan Africa.
Phenomenon of interest	Studies focusing on perceived barriers and facilitators to formal and informal mental health help-seeking behaviours	Studies not examining perceived barriers/facilitators to mental health help-seeking
Population	school-going adolescents (aged 10–19 years).	Studies not involving school-going adolescents aged 10–19
Study design	Studies utilise qualitative research methods such as interviews, focus groups, qualitative content analysis or ethnographic observations.	Quantitative-only studies, studies that did not employ qualitative methods, and those
Publication type	Peer-reviewed full-text articles published in English	Grey literature, non-peer-reviewed sources, non-English publications.
Time frame	Published from 1990 onwards	Published prior to 1990

#### Study selection and screening process

We initially imported the search results into EndNote 20 (Bramer and Bain, 2017; Fulbright and Evans, 2024), whose duplication function was used to identify and eliminate duplicate records.

Four expert reviewers, including the lead author and three academic researchers with MH expertise (AIK, TC, RP and KAP), independently screened the identified articles. Each reviewer assessed the relevance of the articles to the review aim. We enhanced this method by manually searching the reference lists and recording missing citations for important papers in addition to the database search. After the initial title screening, two reviewers (AIK and RP) independently screened the abstracts of the selected studies. Articles approved by at least one reviewer proceeded to full-text evaluation.

Endnote supported the import of all retrieved records and the removal of duplicates at the beginning of the screening process (Ivey and Crum, 2018). The deduplicated library was then transferred to Rayyan to support blinded screening, manage reviewer decisions and record conflicts during title, abstract and full-text assessments (Ouzzani et al., 2016). A custom-made *pro forma* in the form of an Excel spreadsheet (Gibbs et al., 2022) captured the final screening outcomes, documented exclusion reasons and organised the dataset required for PRISMA flow reporting.

### Search results

A comprehensive initial search across six databases, including CINAHL (*n* = 125), PubMed (*n* = 1,054), ERIC (*n* = 105), PsycINFO (*n* = 112), SCOPUS (*n* = 509) and Google Scholar (*n* = 51), yielded 1,905 citations. After importing into EndNote, 298 duplicates were identified and removed. The remaining 1,607 records were screened using a combination of Rayyan (Ouzzani et al., 2016) and EndNote relevance filters. These flagged 267 records as ineligible based on the predefined exclusion criteria (*e.g.*, off-topic titles and lack of relevance to the review question). Another 31 records were removed for various reasons, such as the article format being an opinion piece, full text not accessible despite email correspondence to the author or non-English language. This left 1,309 records for the title and abstract screening.

**Figure 1.**
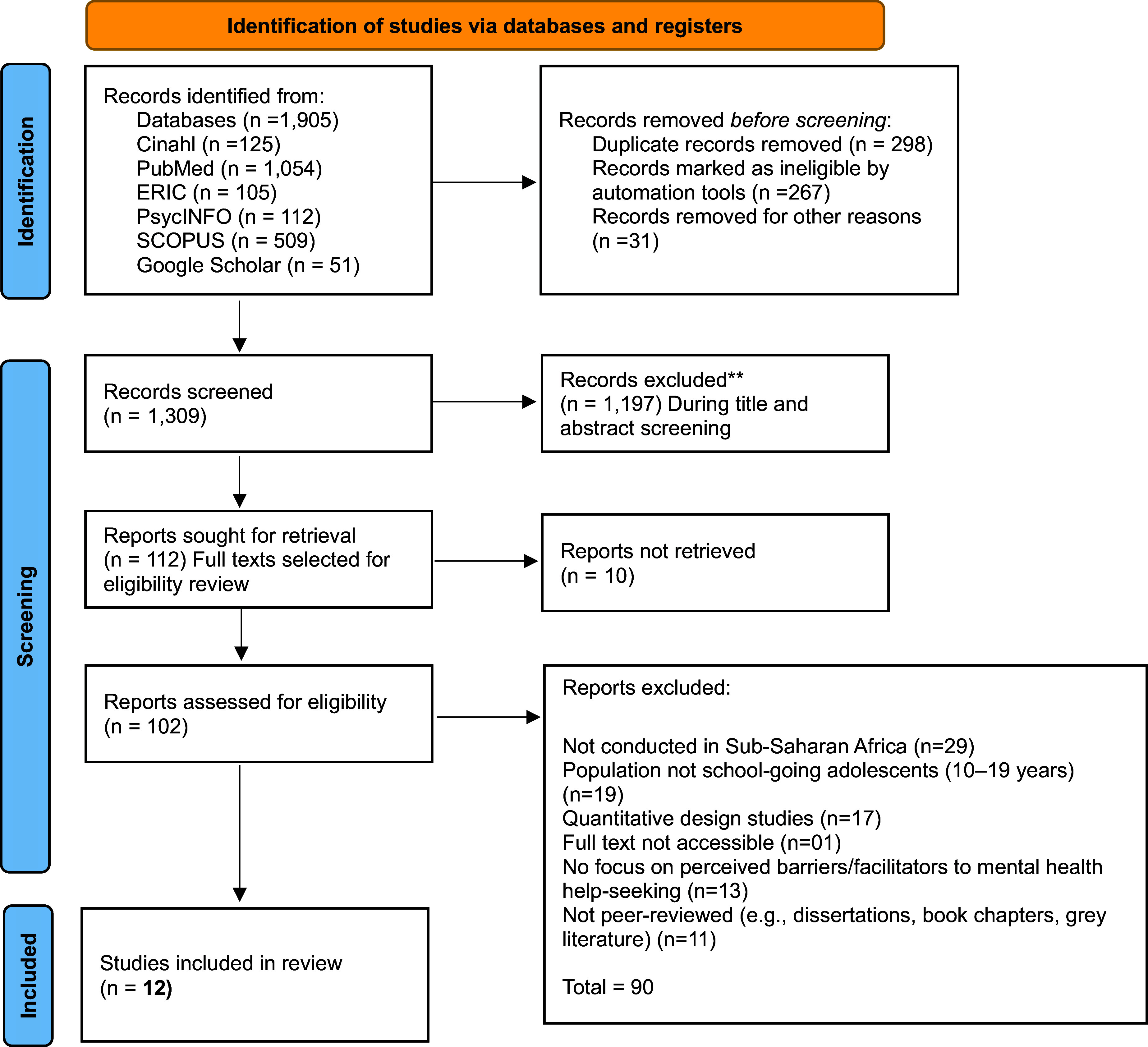
PRISMA diagram for study selection. Figure 1. long description.

Through manual screening, 1,197 records were excluded for not meeting the review’s core criteria, such as population mismatch, irrelevant outcomes and ineligible study designs. Subsequently, 112 full-text articles were retrieved for a detailed eligibility assessment. Of these, 90 articles were excluded for the following reasons: not conducted in SSA (*n* = 29), not involving school-going adolescents aged 10–19 (*n* = 19), not addressing perceived barriers or facilitators to MH help-seeking (*n* = 13), use of a quantitative methodology (*n* = 17), full texts inaccessible despite retrieval efforts (*n* = 01) and not peer-reviewed (*n* = 11). Twelve articles satisfied the requirements and were included in the final analysis. The PRISMA flowchart (Figure 1) visually represents the study selection process (Table 3).

**Table 3.** Characteristics of included studies and their contribution to the review question (12) Table 3. long description.

Title	References	Country/study setting	Participants (n; Type)	Study design	Data collection	Data analysis	Contribution to review question
Mental health issues of school-going adolescents in high schools in the Eastern Cape, South Africa	Mfidi (2017)	South Africa (Eastern Cape; High Schools)	15 participants (7 teachers; 8 school health nurses)	Qualitative descriptive exploratory design	Semi-structured interviews; Focus Group Discussions	Content analysis	Highlights institutional barriers to formal help-seeking, including limited staff training, unclear referral pathways and resource shortages within schools. Indicates that trained and responsive school health personnel may facilitate the early identification and referral of distressed adolescents.
Help-Seeking: A Qualitative study of help-seeking behaviours of students in public secondary schools in Northeast Nigeria	Abdulsalam et al. (2023)	Nigeria (Public Secondary Schools, Northeast region)	24 students (aged 18 years and above)	Qualitative study	In-depth interviews; Focus Group Discussions	Thematic analysis	Identifies stigma, confidentiality concerns and gendered expectations as barriers to formal help-seeking. Indicates that peer support, trusted adults and improved service awareness facilitate both informal and formal help-seeking.
Adolescents’ experience of stigma when accessing school-based PTSD interventions	van de Water et al. (2018)	South Africa (School-based PTSD intervention setting)	10 trauma-exposed adolescents	Qualitative study	Semi-structured interviews; Focus Group Discussions	Thematic analysis	Demonstrates how enacted and anticipated stigma within school environments discourages engagement with formal mental health services. This highlights the importance of confidentiality, peer perception and the normalisation of support in facilitating sustained participation in school-based interventions.
What should a universal school-based psychoeducational programme to support psychological well-being among children and young people in South Africa focus on, and how should it be delivered? A multi-stakeholder perspective	Coetzee et al. (2022)	South Africa (School settings)	66 (students; parents; teachers; school counsellors)	Qualitative multi-stakeholder study	Semi-structured interviews	Thematic analysis	Highlights the structural and relational conditions that shape engagement with school-based mental health programmes, including confidentiality practices, parental involvement and the perceived relevance of content. Identifies inclusive programme design and stakeholder engagement as facilitators of acceptability and uptake within school settings.
Barriers and facilitators of child and guardian attendance in task-shifted mental health services in schools in western Kenya	Meza et al. (2020)	Kenya (Western Kenya; School-based task-shifted mental health services)	36 (teachers; community health volunteers)	Qualitative study	Semi-structured interviews	Thematic analysis	Identifies logistical constraints, caregiver engagement and stigma as barriers to sustained attendance in school-based mental health services. Highlights task shifting, school-based delivery and community involvement as facilitators of service access and uptake.
Student, teacher and caregiver perceptions on implementing mental health interventions in Ugandan schools	Carlson et al. (2021)	Uganda (School-based mental health intervention settings)	59 (students; teachers; caregivers)	Focused ethnography	Focus Group Discussions; In-depth interviews	Framework analysis	This study supports the QES themes of trust, confidentiality and school capacity, showing that caregiver attitudes and institutional readiness shape adolescents’ engagement with formal services.
Knowledge, attitudes and uptake of mental health services by secondary school students in Gweru, Zimbabwe	Khombo et al. (2023)	Zimbabwe (Secondary Schools, Gweru)	15 secondary school students	Qualitative study	Semi-structured interviews	Thematic analysis	This study supports the QES themes on mental health literacy and stigma, showing that limited knowledge and negative attitudes constrain service uptake, whereas improved awareness and positive perceptions facilitate formal help-seeking.
Mental health difficulties, coping mechanisms and support systems among school-going adolescents in Ghana: A mixed-methods study	Addy et al. (2021)	Ghana (Senior High Schools)	53 qualitative participants (students; teachers)	Convergent mixed methods (qualitative component extracted)	Focus Group Discussions; In-depth interviews	Inductive and deductive thematic analysis (ATLAS.ti)	Supports QES themes on informal support systems and sociocultural norms, showing reliance on peers and family, limited formal service engagement and structural constraints within school environments.
Practices and challenges of counselling in selected senior high schools in Accra, Ghana	Panford-Quainoo et al. (2024)	Ghana (Senior High Schools, Accra)	15 school counsellors	Qualitative study	Interviews with school counsellors	Thematic analysis	This study highlights systemic challenges within school counselling provision, including role conflict, work overload, resource deficits and negative perceptions of counselling, which reduce formal help-seeking uptake among adolescents.
Exploring adolescent learners’ perceptions of mental health and behavioural needs in a rural school setting	Mukuna (2025)	South Africa (Thabo Mofutsanyana district; rural high school)	8 adolescent learners from a rural high school	Qualitative study	Narrative interviews with adolescent learners	Narrative analysis	Supports QES themes on mental health literacy and problem recognition, showing that limited awareness and rural context perceptions constrain formal help-seeking, while peer understanding and community-aligned support highlight potential facilitators.
Adolescent learners’ attitudes towards mental and behavioural health needs at a rural high school	Nkosi (2025)	South Africa (Rural high school; Thabo Mofutsanyana district)	8 adolescent learners	Qualitative interpretive phenomenological study	Semi-structured interviews	Narrative analysis (within phenomenological design)	Reinforces QES themes on mental health literacy and stigma, showing that limited understanding, negative attitudes, academic pressures and peer influence constrain formal help seeking, whereas positive peer norms and awareness support informal help seeking.
Parental involvement in school-based mental health interventions for young people in low-resource settings: A qualitative study from Zimbabwe and Ghana	Mushonga et al. (2025)	Zimbabwe (Harare) and Ghana (Navrongo)	Stakeholders, including parents, teachers, school-based mental health providers and young people aged 15–24 in Zimbabwe and 15–18 in Ghana	Qualitative study	Semi-structured in-depth interviews, key informant interviews and focus group discussions	Inductive thematic analysis	This study strengthens the QES themes on family influence by showing that parental engagement facilitates access to school-based services, whereas privacy concerns and family dynamics constrain formal help seeking.

### Data extraction and study characteristics

The researchers conducted a systematic data extraction procedure after the 12 studies were selected for the QES. The research data extraction template included study characteristics (author name, publication year and country/setting) and methodological features (design approach, sampling method, data collection and analysis approach). Participant details and relevant first- and second-order constructs (direct participant statements and researcher interpretations) that matched the research question were also included.

Across the 12 included studies, qualitative interview-based designs predominated, with thematic analysis being the most employed analytic approach. The studies spanned Southern, West and East Africa, including South Africa, Ghana, Kenya, Nigeria, Uganda and Zimbabwe. Earlier studies primarily centred on adolescent perspectives, whereas more recent work incorporated multi-stakeholder and implementation lenses, reflecting a shift towards systems-level analysis. Across contexts, the findings consistently converged on institutional capacity constraints, confidentiality practices, stigma and family engagement as central determinants of formal and informal help-seeking within school settings.

The authors RP and AIK assessed all 12 studies. The dual review process entailed a comparison of the extracted data fields with the original study texts to verify their completeness and precision while maintaining consistent interpretation. The reviewers resolved differences in participant categorisation and thematic content interpretation through consensus discussions. The minor revisions involved improving the wording of the extracted themes to more accurately represent the participants’ meanings and correcting methodological descriptor inconsistencies.

### Quality and risk of bias assessment process

AIK and RP evaluated the reporting quality of the 12 papers included in the review. We employed the Critical Appraisal Skills Programme (CASP) checklist (Long et al., 2020; Haile, 2022; Maeda et al., 2023), a widely used instrument specifically developed to assess the reporting quality of qualitative research. The inter-rater agreement between the two reviewers (AIK and RP) was assessed using Cohen’s kappa (Kilpatrick et al., 2024) based on independent ratings of the 12 included studies using the CASP checklist. The observed agreement was 87.5%, and the expected agreement by chance was 56.3%, resulting in a Cohen’s kappa of .714, which indicates substantial agreement.

The CASP checklist consists of 10 essential enquiries, each designed to evaluate distinct aspects of the research. These include a well-defined research purpose, methodological rigour, study design rationale, participant recruitment strategy, data collection methods employed (including explicit consideration of the researcher–participant relationship), ethical concerns and detailed processes undertaken for analysis to ensure analytical validity, transparency and clarity in the findings reported. Consequently, the synthesis included 12 studies with a total of 10 parameters (Table 2). Khombo et al. (2023) did not meet the “clarity of the aims of the research” criterion. However, their study did not fall short on any of the other nine parameters used in this study. As the remaining criteria were satisfied, this study was included in the review. See Supplementary Appendix B for the study evaluation table based on the CASP.

### Thematic analysis, coding and synthesis

We employed an inductive thematic analysis to establish the codes. This method allowed for the emergence of themes from the data rather than being pre-defined (Braun and Clarke, 2023). In NVivo, we sorted and coded the data according to various patterns and themes. This was done by reading and re-reading the primary source data (*i.e.* quotes by adolescents, MH experts and various stakeholders in the selected papers).

### Confidence in the evidence

The review authors, AIK and RP, used the GRADE-CERQual approach to evaluate the confidence levels for each finding in their review. The GRADE-CERQual approach evaluates confidence in the evidence based on four main components: methodological limitations of the included studies, coherence of the review findings, adequacy of the data supporting the review findings and relevance of the included 12 studies to the review question (Lewin et al., 2018). The assessment of each component enabled us to reach a collective decision regarding the overall confidence level for each review finding, which we classified as high, moderate, low or very low. We began by assuming high confidence in all findings, which we reduced to lower levels when substantial concerns emerged in any of the four CERQual components. Both review authors reached an agreement during the final assessment (See Supplementary Appendix D, Table 5).

## Results

### Influence of barriers on mental health help-seeking behaviours

Across the reviewed research, the findings revealed that perceived stigma was the most frequently encountered barrier to accessing MH services (Mfidi 2017; Carlson et al., 2021; Khombo et al., 2023). Other common barriers included low MHL (Meza et al., 2020), cost (Carlson et al., 2021), inaccessibility of services (Meza et al., 2020; Addy et al., 2021), issues related to confidentiality (Addy et al., 2021) and negative past experiences with the provider (Khombo et al., 2023). Parents’ attitudes (Khombo et al., 2023) and academic pressure (Meza et al., 2020) further inhibited adolescents from seeking help for MHPs. Table 4 summarises these barriers.

**Table 4.** Barriers to mental health help-seeking Table 4. long description.

	Barrier Theme	Subthemes	Number of Studies
1	Perceived stigma	Social/public stigma; cultural stigma	12
2	Gender norms as a barrier to help-seeking	Masculinity norms and stigma around emotional expression; gendered socialisation and structural constraints on help-seeking	9
3	Mental health literacy (Knowledge, misconceptions and awareness gaps)	Poor recognition of MHPs; limited knowledge and misbeliefs about self-help and professional support options; negative attitudes hindering MHP recognition and help-seeking; limited knowledge of how to access mental health information	11
4	Privacy, trust and confidentiality concerns with MH professionals	Fear of confidentiality breaches; inadequate counselling environments and lack of privacy; distrust in mental health professionals	7
5	Lack of accessibility and availability of MH services	Distance to services; lack of mental health professionals; inadequate resources	9
6	Family and parental attitudes, peer influence and alternative support	Family control and restriction of help-seeking; preference for informal and relational support networks; cultural and normative beliefs shaping help-seeking	7

### Theme 1: Perceived stigma

All studies reviewed indicated perceived stigma as the most frequently cited barrier to MH help-seeking behaviours among school-going adolescents. Stigma was conceptualised across four interrelated dimensions: social/public stigma (negative reactions from others), anticipated stigma (fear of being judged if one seeks help), self-stigma (internalised shame and self-blame) and cultural stigma (beliefs linking mental distress to moral, spiritual or supernatural explanations). Adolescents who encounter stigma related to MH are less likely to acknowledge their potential MHPs and seek formal or informal help for their MH. This collectively creates an environment in which seeking help for MHPs is met with shame, fear and reluctance to seek help.

The pervasiveness of stigma and cultural standards has contributed to an atmosphere in which confronting problems related to MH elicits feelings of dread and shame. For example, in some schools and communities, adolescents who express emotional distress or seek MH support risk being labelled as weak or disobedient, especially where strength and emotional control are linked to respectability and maturity.

The stigma surrounding MH topics is often reinforced socially by peer groups, either explicitly or implicitly. Adolescents often face severe social/public stigma from their families, peers and communities in SSA (Carlson et al., 2021; Khombo et al., 2023). This stigma usually stems from negative opinions and false beliefs about MHPs themselves, such that the fear of being ostracised or labelled as “weak” or “crazy” prevents adolescents from acknowledging their MHPs and seeking help. Evidence from one included study indicates that adolescents encounter overtly negative peer reactions to MH difficulties, as reflected in the following account: *“Their response is unkind, and their behaviours can be shameful. They show emotions of fear and hate*…” (Nkosi, 2025, p. 27), demonstrating a deep-rooted fear of judgement.

Similarly, another study reported that female adolescents avoid disclosure due to fear of being labelled or judged: “*When you let people know what you are going through, they will think you are mad. I just keep to myself*” (Addy et al., 2021, p. 12). This demonstrates that adolescents, in their bid for acceptance, are sensitive to how their peer group may react, thus inhibiting them from seeking help (van de Water et al., 2018). For example, one study reported that peers often respond with ridicule or exclusion: “*People would ‘[not] talk to them. They make fun of them,’ say, ‘you are crazy’ and ignore them, or ‘judge them.”* (van de Water et al., 2018, p. 1091).

Adolescents are often worried about the disclosure of their MHPs, as it can lead to rumours and stigma among the public. For example, one study reported the following: *“…I heard people in the community whispering my name about something I had said in the session, and it broke my trust…”* (Khombo et al., 2023, p. 5).

A participant in another study reported a similar stigmatising experience: “*I heard students saying they were making fun of themselves, and they said, ‘you, you have a mental problem, go and talk to the counsellor, go and see the counsellor’.”* (Panford-Quainoo et al., 2024, p. 190). Overall, social and public stigma cumulatively contribute to an inhospitable atmosphere for adolescents, in turn discouraging them from seeking MH services. The widespread presence of these stigmas intensifies the lack of communication with others about one’s MHPs, further exacerbating isolation and the potential for more severe MHPs.

In addition to social and public stigma, SSA communities are confronted with cultural stigma that affects adolescent help-seeking (Khombo et al., 2023). Cultural stigma refers to the beliefs and views that connect MHPs with weakness or failure embedded within a cultural context (Codjoe et al., 2021; Ojagbemi and Gureje, 2021). In many SSA cultures, MHPs are often seen as having a lack of will or a spiritual problem that causes families and communities great shame and humiliation. One secondary school student in a study from Ghana stated, *“When you start hearing voices, it’s only the spiritualist like fetish priests who can help you”* (Addy et al., 2021, p. 12). Additionally, another secondary school student perspective reflects this noting that*“…these psychotic mental illnesses are said to be caused by evil spirits or to be a kind of retribution from supernatural spirits or beings” (*Nkosi, *2025, p. 28).* A participant in another study reported, “*My family… were like, ‘Counselling? Really?’… ‘Counselling is for White people only.*’” (van de Water et al., 2018, p. 1092). As Brouwers (2020) propose, culture can also breed silence among MHPs.

### Theme 2: Gender norms as a barrier to help-seeking

Gender norms play a significant role in adolescents’ decisions to seek MH help (Campbell et al., 2021; Jörns-Presentati et al., 2021). Studies have indicated that female students are generally more willing to seek professional help than their male counterparts (Khombo et al., 2023). This difference stems from societal norms that label emotional expression as a weakness, especially among boys (Meza et al., 2020; Coetzee et al., 2022). In many cultures, boys are expected to be stoic, making it difficult for them to acknowledge psychological distress or to seek help (van de Water et al., 2018; Carlson et al., 2021; Abdulsalam et al., 2023).

In contrast, girls are socialised to be more open about their feelings and encouraged to seek support when needed (Khombo et al., 2023). Evidence from one included study illustrates how prior exposure to counselling can reinforce help-seeking among female students:*“…female participants use more emotion-focused strategies, such as seeking emotional support from others than male participants*” (Khombo et al., 2023, p. 3).

However, male students often avoid MH services due to fear of losing their dignity. For example, one study reported: “*Seeking counselling is for women; men bottle their issues lest they lose their dignity.*” Similarly, a participant from another study reported, *“As a male, this thing of going to see someone to cry and talk to is taboo; it takes away my masculinity*” (Khombo et al., 2023, p. 5). Similarly, another study reported a participant who stated, “*I think the girls and boys experience things that they wouldn’t necessarily want to say in front of the other, or the boys are going to be too cool to say in front of the girls…”* (Coetzee et al., 2022, p. 196). Societal pressure to conform to masculine ideals makes it harder for boys to seek help.

To further illustrate the impact of gender norms as a barrier to help-seeking, Abdulsalam et al. (2023) found that both gender and age play a role in whether adolescents in Northeastern Nigeria seek help for MHPs. These patterns are further influenced by the interaction between cultural or religious norms and the student’s gender. For example, one study reported a participant who stated feeling uncomfortable when a young man was selected as a counsellor: “*I don’t feel like going for counselling there. Our counsellor is a young male counsellor. In Islam, an unmarried girl should not sit alone with a man not from her family because something bad may happen between them.*” (Abdulsalam et al., 2023, p.12). Male counsellors also face difficulties due to religious beliefs. One counsellor noted, “*There are insinuations and allegations if you are a young, unmarried chap as a counsellor. Because you sit with students, especially females, people suspect you, not that you are doing your counselling work”* (Abdulsalam et al., 2023, p.12). Identifying and managing gender dynamics is necessary to improve adolescents’ MH help-seeking behaviours.

### Theme 3: Mental health literacy (knowledge, misconceptions and awareness gaps)

Due to low MHL, defined as “knowledge and beliefs about MHPs which aid their recognition, management or prevention” (Jorm et al., 1997, p.182), adolescents do not always seek help for MHPs (Amone-P’Olak et al., 2023; Ma et al., 2023; Clough et al., 2024). Four main components affect adolescents’ knowledge and behaviour regarding MH, including: poor recognition of MHPs; limited knowledge and misbeliefs about self-help and professional support; negative attitudes hindering MHP recognition and help-seeking; limited knowledge regarding how to access MH information.

Often, adolescents misunderstand or mislabel their MHPs due to, for example, parents mislabelling an adolescent’s MHPs as mere obstinacy or wilfulness. Many believe that their distress is temporary or normal and do not believe that it is a mental illness. One study reported a participant who highlighted, “*Some of [the guardians] would not come… they did not understand the value of psychological treatment (PT).*” (Meza et al., 2020, p. 6). Another study reported a participant who revealed, “*Parents wouldn’t know what their children are going through. They are not even aware. They will just say it’s stress. They just take it for granted. So, they should attend those meetings*” (Mushonga et al., 2025, p. 7). This barrier was also found in teachers, as one student stated, *“My teacher thinks I am lying about it [attending counselling]. Because I am too naughty, I don’t go to school*” (van de Water et al., 2018, p. 1092).

Some adolescents reduce MH to only stress and emotional control, as one study reported the following: “*Mental health is when you are stressed or anxious about certain things (s) in your life. Some learners may be able to handle them, some may not. When they do not, they may not behave well.*” (Mukuna, 2025, p. 15). Another study reported a teacher who confirmed the above narrative when she stated, “*sometimes we may mistakenly label the adolescents as ill-disciplined or moody whereas it may be signs of an emotional problem*” (Mfidi, 2017, p.8). When distressed, adolescents usually do not seek help because they do not recognise that something is wrong with them.

Furthermore, adolescents do not often understand MHPs and their development. As a result, when they experience a MH concern, they may interpret these symptoms as personal weakness and feel compelled to manage them independently rather than seek support. This internalisation can interfere with their ability to concentrate and participate in academic activities, as distress remains unaddressed. For example, one study reported that distress can disrupt attention and engagement in classroom settings “:*If that child is sitting with that blow… in class… and that teacher is explaining something here on the board and that child’s… attention is here with the pain… or with that person who hurt him*…” (Coetzee et al., 2022, p. 194). Similarly, another study reported that limited awareness contributes to dismissive attitudes towards MH: “*Learners at my school are nonchalant towards issues like mental health and behavioural problems. They are not well educated or well informed about these issues*…” (Nkosi, 2025, p. 30). In the absence of knowledge about the risk factors and causes, adolescents may fail to understand the gravity of the disorder and the urgent need for help to address it.

Adolescents often do not know where to go or how to help themselves effectively when MH issues arise. One study reported: “*If I need some to talk to, I talk to my boyfriend who is always there to offer me advice and comfort when I am stressed, so I don’t think I need a counsellor or a psychologist to deal with my problems*” (Khombo et al., 2023, P.6). Many would rather seek informal support than recognise and use self-help strategies or professional resources.

One major aspect of MHL is awareness of the professional help available. Many adolescents do not know how to seek help when they need it. One study reported that adolescents often experience uncertainty surrounding the help-seeking experience, stating: “*The clinic… they are going to ask too many questions… They would just have told me to ‘just get it out of my mind’… and then told me to go home.”* (van de Water et al., 2018, p. 1092). This lack of knowledge and subsequent mistrust was further reflected in another study where a participant expressed the following:

*I do not trust doctors… They look nice, but no, I do not trust them… I was going toward a social worker, a real social worker. Not doctors. Even if they come with a social worker, I would ask them what company you are working for. I am going to ask her a question… If I have to Google about it, I will Google. I must make sure where I am”* (*van de Water et al., 2018*, p. 5).

The lack of knowledge about available professionals contributes to adolescents’ unwillingness to seek prompt help due to a lack of understanding and trust. Adolescents often remain hindered by negative attitudes from both themselves and providers and an inability to recognise signs and symptoms of MHPs. Most adolescents blamed their MHPs on themselves instead of considering them a medical issue. One study reported, “…*they will try for a day or two, like I said, but their attitude is already ‘it’s [the programme] not going to work, the children are broken, they come broken from home, so what should I now fix?’”* (Coetzee et al., 2022, p. 193).

Adolescents require further guidance when searching for correct MH information. One school administrator indicated that teachers and other staff lacked knowledge about MH in general: “*Some teachers are unable to recognise and seek help for their learners’ MHPs due to lack of specific training on mental health*” (Mfidi, 2017, p. 10). Another study reported, “*I may have studied psychology, but at times we are not skilled to deal with some of these social, emotional, and behavioural problems. We just have to refer them*” (Mfidi, 2017, p. 7). This perception is further illustrated by the following statement by a participant in another study: “*The thought of talking about my life might bring up some problems I thought I had overcome, and that could increase my stress. So, whenever I hear about mental health issues, I just avoid them*” (Abdulsalam et al., 2023, p. 8).

This lack of awareness about existing MHPs applies to the school environment as well. Usually, teachers are the first point of contact for students experiencing MHPs, but teachers often do not receive adequate MH training. For example, in one study, a teacher noted, “*I can help. I just do not know what it looks like when a child needs help*” (Meza et al., 2020, p. 8). Similarly, another teacher remarked, “*I feel unprepared to handle mental health issues among students because I haven’t received any training in this area*” (Mfidi, 2017, p.8). This lack of awareness from teachers, who are often the first critical point of contact for struggling students, further hinders student awareness, “*I have never been to counselling before, so I never knew what counselling was about*” (van de Water et al., 2018, p. 1093).

This MHL gap also applies to parents who may not be equipped to help, depending on their MHL. For example, A teacher in one study described that some caregivers are *“…people who do not know how to handle their emotions themselves so they cannot help children*…” (Coetzee et al., 2022, p. 6). Some adolescents and their parents may also not fully appreciate the usefulness of MH services. A study reported a participant who indicated a reluctance to seek professional help, stating: “…*I don’t want to hear about psychological issues or having to go and see a counsellor, it really makes me sick.”* (Khombo et al., 2023, p.5).

Many adolescents further believe that if their friends or partners support them, they do not need to seek help from professionals. This is reflected in one study: “*I talk to my boyfriend…so I don’t think I need a counsellor*” (van de Water et al., 2018, p. 8), further illustrating the preference for informal support over professional help.

Collectively, these studies revealed that participants associated seeking help for MHPs from a MH practitioner with inferiority or weakness. These perceptions and the lack of knowledge and awareness collectively inhibit effective help-seeking behaviours, indicating a need for more MHL.

### Theme 4: Privacy, trust and confidentiality concerns with mental health professionals

Concerns about privacy and confidentiality are significant barriers to adolescents seeking MH services in SSA (Mutahi et al., 2022; Hlophe et al., 2023). Adolescents frequently express fear that their disclosures might be revealed to others, such as teachers, parents or peers (Yao et al., 2021). For example, one study documented the following “:*I know the school has a counselling unit. But ahhh… I will not go there. I am shy of the teachers*” (Addy et al., 2021, p. 12).

The physical setting of counselling offices exacerbates these problems. Open or poorly designed spaces are perceived as unsafe, discouraging help-seeking behaviours. Inadequate infrastructure, such as counselling offices divided by plywood, creates an environment where adolescents feel exposed. One study documented the following: “*Our counselling office is too open. They used plywood to divide the library and the office; I feel people hear what I discuss. To me, the place is not ok; it is not worth it*” (Abdulsalam et al., 2023, p. 10). Another study further described the experience of exposure: “*It’s like the whole world is watching me; I feel I was doing something wrong… worried since the room is not hiding*” (Abdulsalam et al., 2023, p. 10). Alarmingly, these concerns are not unwarranted, as adolescents report a general lack of privacy related to MHP help-seeking: “*They would call our names on the intercom, and all of the kids would be like ‘where are you going, what’s going on there?*’” (van de Water et al., 2018, p. 1094).

Trust in the MH care provider is another critical issue. Adolescents often report negative experiences with professionals, leading to a lack of faith in counselling services. One study presented the following account:

……*I do not trust anyone. If I do not trust my mom and dad, why would I trust someone I am not even related to? I have been disappointed by people I trust in my life and trusting someone I do not know is not something I will ever do. I tell my stories to God whenever I face challenges*… (Khombo et al., 2023, p.5).

A professional in one study confirmed the above by stating *“From my encounter with those who came to me, initially, they were hesitant because of some previous experiences that students would share; they went to see a counsellor, and the next moment, somebody had heard their issues.*” (Panford-Quainoo et al., 2024, p. 190). Another shared how a breach of confidentiality during group therapy destroyed their trust:

…*I have been to group therapy before, and a few days after I attended a session, I heard people in the community whispering my name about something I had said in the session, I will never trust anyone in the counselling session, including the counsellor*… (Khombo et al., 2023, p.5).

Adolescents also doubt counsellors’ capacity to understand their issues: “*I do not believe the counsellors understand our problems; they just follow a script*” (van de Water et al., 2018, p. 1091). One study provided the following account related to counsellors providing services internally within the schools: “*I don’t think it should be internal because it helps them if it’s someone from outside… it’s delicate… not something that children [are] necessarily going to share with a teacher”* (Coetzee et al., 2022, p. 195). This reveals another layer, such that students doubt that school counsellors will keep their MHPs private. Another study provided the following account of a student’s concerns related to counsellors sharing information: “*She is going to speak about it with… other counsellors*” (van de Water et al., 2018, p. 1091). Such perceptions, combined with stigma, encourage students to “sneak in” for counselling, as observed by one teacher–counsellor in another study: “*Students who come for counselling don’t feel at ease… more like sneaking in, watching when to come… like it’s a taboo*” (Abdulsalam et al., 2023, p.10).

According to the adolescents, such perceptions of confidentiality breaches profoundly impact their trust in MH services (Haraldsson et al., 2022; Kip et al., 2022). Thus, it is imperative to ensure that counselling takes place in a secluded location and that the counsellor faithfully adheres to confidentiality rules to build and keep trust with adolescent clients. Students must be informed about these confidentiality protocols ahead of sessions.

### Theme 5: Lack of accessibility and availability of mental health services

Lack of access to MH services is another major barrier to help-seeking behaviours among adolescents, particularly in rural SSA (Sarikhani et al., 2021; Mindu et al., 2023). Owing to geographic proximity, adolescents in rural educational institutions often lack access to qualified MH professionals, such as counsellors or counselling psychologists. An administrator from one study stated, “*Many rural schools have difficulty adopting and implementing school mental health systems… This is caused by insufficient specialised mental and behavioural health personnel such as school counsellors, psychologists, and social workers*” (Nkosi, 2025, p. 27). Similarly, an administrator from another school stated, “*During the rainy season, heavy rain significantly increased transportation fees and made roadways to school impassable, posing a barrier to students and their parents’ attendance*” (Meza et al., 2020, p.8).

In addition to issues associated with counselling accessibility at rural schools, another study reported institutional capacity constraints: “*Well, I think the challenges, one is the fact that you’re playing a double role; you’re a counsellor, you’re a teacher. So, when you are in the classroom, and somebody needs you for counselling and you’re not available*” (Panford-Quainoo et al., 2024, p. 190).

Financial constraints further exacerbate accessibility-related hurdles. Low-income communities and their respective schools sometimes experience substantial economic adversity, leading to a focus on meeting essential requirements such as food and school expenses rather than prioritising MH care. As one study reported, “*We used to go together with health promoters… But now the transport we use cannot accommodate them*” (Mfidi, 2017, p. 8). Similarly, a participant from another study reported, “*Currently they [school counsellors] are full to the ears… I just feel those children—I think some of our parents do not have the means to get professional help for them.”* (Coetzee et al., 2022, p. 193). Many adolescents cannot afford MH services because of the costs involved, whether for the service itself or for the related expenses, such as transport. Many colleges also do not have adequate counselling facilities, nor educated professionals to ensure a standard of MH care for students. One study illustrated this by stating, “*You could sit in the queue the whole day but not get the help you needed.*” (van de Water et al., 2018, p. 1095). Thus, accessibility and availability issues prevent MH help-seeking among adolescents despite their intentions to do so.

### Theme 6: Family and parental attitudes, peer influence and alternative support

It has been well-established that family and parental attitudes can serve as barriers to the MH help-seeking behaviour of adolescents. The attitudes of some parents become a barrier, blocking their children from seeking support. For instance, one study reported a participant who remarked, “*Pressure from our peers can impact us negatively, including the abuse from guardians, parents… Abuse from our parents impacts us a lot, and it is not easy to talk about it…”* (Nkosi, 2025, p. 30). Despite the potential for negative attitudes, parents and families still provide alternative sources of emotional and financial support for adolescents, who often seek comfort from family instead of MH professionals. This is reflected in the following quote: *“Parents are the first source of life to the child in terms of love and care. This is why students are homesick when they need advice… to discuss their problems closely, a kind of allegiance.*” (van de Water et al., 2018, p.6). Similarly, another study reported, “*They feel it is their duty, a natural duty… they feel they are more obliged as parents or family to advise and guide the student. This perspective affects the sensitivity of the child; nearly in everything, including getting help*” (van de Water et al., 2018, p.6).

Siblings and extended families also play a significant role in help-seeking activities. Adolescents often discuss issues with their older siblings when they do not wish to reveal perceived confidential information to their parents. According to one study, older siblings counselled younger ones on choosing a career, higher institution and gender-related issues such as relationships and social expectations (Abdulsalam et al., 2023). However, judgement from parents about MH can discourage treatment: “*I don’t think parents truly understand what mental health issues entail. Mine don’t,”* an adolescent remarked (Carlson et al., 2021, p. 8).

During adolescence, when we rely on our social networks, peers may also have considerable influence over one another, as they have established trusting relationships. Some peers function as a barrier to help-seeking; for example, as one participant shared, “*I was embarrassed to go to therapy, especially when my school friends asked about it”* (van de Water et al., 2018, p. 1092), whereas other peers function as an informal help-seeking pathway. For instance, one adolescent reported, “*My boyfriend is the one who comforts me because I do not trust anybody*” (van de Water et al., 2018, p.1092). According to Khombo et al. (2023), most adolescents prefer informal support systems over professional services. One of the main reasons for this is the stigma associated with using professional services.

A preference for familiar connections may subsequently result in reduced engagement with supportive services (Khombo et al., 2023). As one participant noted, “*If I need some to talk to, I talk to my boyfriend who is always there to offer me advice and comfort… I don’t think I need a counsellor or a psychologist to deal with my problems.”* (Khombo et al., 2023, p.6).

Some parents prioritise academic and other responsibilities over MH, which may further discourage adolescents from seeking help for MHPs. As reported in one study, a student presented the following account, “Because some of them are the ones who ill-treat us. If they knew that we are getting help somewhere, they might even hate us. That would become a disadvantage to us.” (Mushonga et al., 2025, p. 10). Such an attitude of overlooking MHPs can fuel the notion that one’s problems may not be serious, so the adolescent avoids professional help. Another study reported a student’s account who shared their experience of their parents’ hurtful and unsupportive behaviour during a traumatic incident, stating:

*[Parents] do not expect the bad from their family … they do not really care. So, it was difficult for me to speak to people about [the traumatic incident] … My daddy went to confront [the perpetrator], and then he denied everything, and my mummy said “ I must not press charges because it is my auntie’s only child” … It felt like no one cared … [when I told my mom and gave the forms and stuff] she just looked at me and then she was like, are you sure it was him?* (*van de Water et al., 2018*, p.1092).

Religious beliefs and practices are another factor affecting the help-seeking behaviours of school-going adolescents. Some students draw upon their religious communities or spiritual tools instead of counselling for support. According to one study, a participant stated, “ *students seek spiritual guidance if they sense the matter is between them and God… strictly spiritual* “(Abdulsalam et al., 2023, p. 1305). This sentiment was echoed by another study which reported, “*When you start hearing voices, it’s only the spiritualists like fetish priests who can help you*” (Addy et al., 2021, p. 12).

### Facilitators of mental health help-seeking behaviours

The reported facilitators of MH help-seeking among adolescents included supportive family dynamics, positive peer influence, accessible informal networks and cultural or community resources (see Table 5).

**Table 5.** Facilitators of mental health help-seeking Table 5. long description.

	Facilitator theme	Sub-themes	Number of studies
1	Mental health education and literacy enhancement	Promotion of mental health literacy; access to information and resources; integration into the school curriculum; parental mental health education	12
2	Supportive school environment or climate	School mental health initiatives; school mental health policies and institutional commitment	10
3	Improved professional services	Availability of trained and specialised mental health professionals; confidential, gender-sensitive and culturally appropriate care	8
4	Family, community involvement and peer support	Family communication and emotional support; peer support and shared coping; community-based support and outreach	8
5	Improved service accessibility and affordability	Policy and financial investment in mental health services; school-based integration of mental health services	6

### Theme 1: Mental health education and literacy enhancement

All 12 studies identified increased awareness and MH education as the main driver of help-seeking behaviours among school-going adolescents in SSA. When adolescents receive MH information that is both clear and culturally relevant, they display higher rates of early symptom recognition and are more willing to pursue help while talking openly about their MHPs. Adolescents are also more likely to seek help when MH education is integrated into life skills training by teachers and when peer leaders facilitate open discussions with students. Promotion of MHL at the school and community level was identified as an important facilitator for enhancing help-seeking behaviour among school-going adolescents (Meza et al., 2020; Carlson et al., 2021; Coetzee et al., 2022). To illustrate this, one participant (female caregiver) noted:

*I am of the view that if all the stakeholders in the school could be sensitized—what do I mean by the stakeholders? The parents, the children themselves, the teachers, and the people who manage the school—about how children can be handled. I think it can help the children to have good mental health. (Carlson et al., 2021*, p.6).

Another study noted the benefits of MHL, stating that “*Learning about mental health in school made it easier to talk about my problems*” (Carlson et al., 2021, p. 7). Similarly, interdisciplinary collaboration within school settings was also identified as a mechanism for strengthening MH support, particularly through coordinated involvement of teachers, parents and school leadership. As one school stakeholder emphasised, “*The promotion of mental health through interdisciplinary collaboration in schools was also deemed essential*” (Mfidi, 2017, p. 8).

In addition to increasing MHL as a facilitator of help-seeking, visibility of MH services for adolescents helps to clarify available resources and enhance the likelihood of their utilisation (van de Water et al., 2018). For example, flyer distribution, community programming and information dissemination through the internet may enhance awareness of MH services for school-going adolescents. As one student indicated, “*It was through the support and information I received during the trial that I learned about PTSD and the available treatments. This knowledge motivated me to seek help when my symptoms worsened”* (van de Water et al., 2018, p. 1091). Another participant remarked on the helpfulness of peer education, *“Peer education programme undertaken by the provincial department is not regularly done, but it does help”* (Mfidi, 2017, p. 7). Integrating MH education into the school curriculum was noted as another critical facilitator of help-seeking (Carlson et al., 2021; Coetzee et al., 2022). Incorporating MH education into the curriculum gives adolescents a platform to express themselves and encourages them to seek help for MHPs. One study reported, “*School counsellors recommended that the content of such a programme should be psychoeducational and in-line with the current school-curriculum.*” (Coetzee et al., 2022, p. 195).

One study provided the following account of a teacher who stated that MH education could be beneficial in preventing suicide ideation and behaviours, suggesting the following: “*Just like we have this sex education thing, I think it [mental health education] should be added because of the way people are even committing suicide today because of some trivial issues”* (Addy et al., 2021, p. 13). Another adolescent noted the benefits of MHL in school, stating “*Learning about mental health in school showed me it’s important to seek help*” (Carlson et al., 2021, p. 8). Therefore, introducing MH education into the school curriculum can help enhance the students’ knowledge and outlook towards MH, which in turn encourages them to ask for help when needed (van de Water et al., 2018; Carlson et al., 2021; Khombo et al., 2023).

Despite issues related to the lack of MH education and literacy, findings showed that education programmes can help families and parents understand the mental wellness needs of adolescents. Workshops and seminars for parents, for example, can create a bridge between home and school activities that further facilitate a positive environment for adolescents. As a study reported, *“The workshops assisted my parents in understanding what I am facing and how I could be assisted*” (Addy et al., 2021, p. 13). Another participant remarked, *“The only way you can get most of the parents is usually when there is a PTA meeting. So that is a way that you can get them and give them that education concerning mental health issues.*” (Mushonga et al., 2025, p. 6). These educational programmes for parents can equip families with the skills to identify and deal with MHPs.

### Theme 2: Supportive school environment or climate

The second facilitator theme that emerged from the QES was a supportive school climate, which can motivate school-going adolescents from SSA to seek help for their MH. A school’s climate is defined as the socio-cultural, psychological, safety and educational environment that stimulates learning, social growth and emotional well-being (Wang & Degol, 2016). Across studies, participants noted that programmes designed to support student MH would be beneficial: “*Teachers and school counsellors explained that a programme like this would help them meet the demand for mental health and behavioural support in the schools”* (Coetzee et al., 2022, p. 193). Among studies of schools where such programmes have been implemented, participants generally report positive feedback, with one noting that it was “*a surprise to find ‘someone out there that cares’*” (van de Water et al., 2018, p. 1094).

Our review further found that a positive school climate is reflected in the fair implementation of rules and regulations, the promotion of norms and values, student engagement, attachment to school and teaching activities that enhance learner agency (Thapa et al., 2013). Schools that offer counselling services and maintain a positive environment can help students to deal with their MHPs (Carlson et al., 2021; Coetzee et al., 2022). Involving MH experts in providing support for students, such as counsellors and educational psychologists, rather than relying solely on teachers, is highly effective (Coetzee et al., 2022). Teachers and parents should serve as the first point of contact and a referral pathway to these specialists.

Our findings also demonstrated that to develop a positive school climate that encourages adolescents to seek help, policies surrounding MH must be created (Coetzee et al., 2022). In schools, where guidance and counselling units are well established, students and teachers understand the importance of strong support systems in promoting adolescent MH (Carlson et al., 2021). One participant in a study stated, “*For me, I see them having a positive view about counselling in general… the trust has been built; freshers learn from those who are already in the system, and those in that system speak well about my office*” (Panford-Quainoo et al., 2024, p. 191). According to another teacher, providing a source of support by actively listening to students’ concerns is also helpful in enhancing school climate:

*…where a child is listened to, whenever a child has a problem, there must be someone to listen even if you cannot act, but just giving a child time makes that child have [better] feelings and will be free with whatever problem will not keep any challenge the child has, he will be able to always to speak out whenever he has a problem”* (*Carlson et al., 2021*, p. 4).

### Theme 3: Improved professional services

Schools require the presence of trained MH professionals, such as counsellors and psychologists, to take care of the MH of adolescents. Students should not receive counselling services from teachers. Rather, teachers should refer students to a MH professional, who can refer them to proper interventions (Panford-Quainoo et al., 2024; Nkosi, 2025). Support for counselling services was noted across studies. For example, a participant from one study noted: “*Having a professional counsellor at school makes me feel like my problems are taken seriously*” (Abdulsalam et al., 2023, p. 1202). This is similarly reflected in another study, “*It was a surprise to find ‘someone out there that really cares’*” (van de Water et al., 2018, p. 1093). These perceptions speak to the faith students have in MH professionals working within school settings, as well as the positive outcomes for students with access to these professionals.

One of the most important components of MH services offered in schools involves the assurance of confidentiality. Our review revealed that assured confidentiality enhances trust, as users feel less at risk of negative judgement, stigma or exposure to their peers and family (van de Water et al., 2018; Meza et al., 2020). For instance, one participant from van de Water et al. (2018) remarked: “*I was relieved to share my secret with the nurse counsellor because she’s not my friend and she’s not part of my family*” (van de Water et al., 2018, p. 1094). Similarly, another study reported, “*I can speak about my issues easily because our school keeps everything private.”* (Carlson et al., 2021, p. 7).

### Theme 4: Family, community involvement and peer support

Family, community and peer involvement encourage school-going adolescents in SSA to seek help for their MHPs (Coetzee et al., 2022). These types of involvement include open communication, peer support and encouragement and community outreach. Open communication within the family was important in helping adolescents to seek MH help. Regular and open discussions within families about MH can be effective in reducing stigma and encouraging adolescents to seek help. As one study reported,

*Where I come from, we do not tell anyone who is outside of the family anything we are going through, both on a personal and family level. Whenever we have challenges in life, we find someone we are close to in the family, we tell them, and we get help….* (*Khombo et al., 2023*, p.5).

In addition to family, a peer participant described offering sustained emotional support:

*If you need somebody to talk to, I am here for you… Even if you go through bad things …. I can help you … Cause me too I was in a very bad space. I will go with you until you finish your counselling* (*van de Water et al., 2018*, p. 1095).

Similarly, a teacher highlighted the positive role of families in adolescents’ help-seeking, stating, “*Parents are the first source of life to the child in terms of love and care. This is why students are homesick when they need advice … to discuss their problems closely, a kind of allegiance* (Abdulsalam et al., 2023, p. 5).

Student-led peer support groups and peer counselling initiatives are also powerful facilitators of MH help-seeking. Specifically, help-seeking programmes facilitate and motivate a culture of seeking help among adolescents and further create a conducive environment for them to relate to and empathise with each other. For instance, a teacher from a study conducted in Uganda revealed:

*For the groups, when they come together and they talk about the problems, first, they will know that they are not alone. Other people have the same problem and then they will also learn how they can be able to handle that problem. [They will] learn from the other friends. When this one happens, what do you do? They can also learn how to cope with their problem from their friends* (*Carlson et al., 2021*, p. 5).

In addition to family and peer support, organisations within the community offer outreach programmes to make MH services accessible. These initiatives help link formal MH services with the community. A representative of one of the local schools revealed in a study how local Non-Government Organisations (NGOs) have been helpful to them by providing free services to students: “*Local organisations run mental health workshops that are helpful to our students, especially to our schools, who are still struggling with having mental health budgets”* (Abdulsalam et al., 2023, p. 8). Culturally appropriate MH services must be ensured through outreach services and appropriate community programmes. These programmes allow adolescents and their families to access the necessary help in their communities, reducing barriers to help-seeking.

### Theme 5: Improved service accessibility and affordability

Adolescents showed increased readiness to seek MH help when they could access affordable services. This QES revealed that students showed increased participation in MH programmes, which were located near their schools or communities, when these services had reduced or no associated costs. Research demonstrated that school-based MH programmes significantly affected students’ willingness to pursue MH support (Carlson et al., 2021; Khombo et al., 2023; Panford-Quainoo et al., 2024). Adolescents encountered fewer logistical and financial obstacles when MH services were integrated into their school environments. We found that policies developed to increase the funding available for MH initiatives in schools and communities have the potential to significantly improve services. Despite this, a lack of focus on the funding of MH interventions limits their success and hinders progress. However, it has been revealed that communities where the government has deliberately supported MH initiatives have yielded positive MH outcomes.

A viable strategy for improving accessibility and affordability of MH services is through their integration into schools. If MH specialists are integrated into schools, they could provide counselling to the students as well as the community. This approach addresses accessibility issues with on-site services, which are cost-effective and easier to use. A participant from one study emphasised the importance of accessible support within schools, stating, “*It’s very necessary… because they don’t have support at home… they need someone or a programme like that to help them.*” (Coetzee et al., 2022, p. 193).

## Discussion

Adolescent MH help-seeking presents a persistent global public health challenge, with evidence across regions indicating that stigma, limited MHL and structural access constraints impede timely engagement with support services. In high-income settings, barriers often centre on individual attitudes, concerns about confidentiality within formal systems and service accessibility. In contrast, help-seeking behaviours in SSA are more deeply embedded within sociocultural norms, family authority structures, religious interpretations of distress and institutional resource limitations. This QES situates SSA-specific findings within the broader global literature while foregrounding the contextual realities that shape how school-going adolescents interpret distress and navigate formal and informal support offered by or in educational settings or systems.

Our extraction of themes identified six barriers to MH help-seeking, including: pervasive stigma, MHL (knowledge gaps, misconceptions and limited awareness of services), gender differences, privacy, trust and confidentiality concerns, lack of accessibility and availability of services and family and parental disapproval. For clarity, the theme “MHL (knowledge, misconceptions and awareness gaps)” is used consistently throughout this manuscript to capture deficits in symptom recognition, misunderstanding of causes and treatment and limited awareness of available services. The term “low MHL” refers specifically to this broader construct and does not represent a separate barrier. Overall, perceived stigma emerged as the most robust barrier to school-going adolescent help-seeking behaviour, which took the form of social stigma as well as self-stigma and anticipated discrimination (Mfidi 2017; Addy et al., 2021; Abdulsalam et al., 2023).

The second most prevalent barrier was low MHL, encompassing knowledge deficits, misconceptions, awareness gaps, along with privacy, trust and confidentiality concerns (Meza et al., 2020; Addy et al., 2021; Coetzee et al., 2022; Panford-Quainoo et al., 2024; Mushonga et al., 2025). These factors contributed to the delayed recognition of symptoms and a lack of awareness of available services. Boys were less inclined to pursue professional help, as gender norms related to masculinity functioned as an influential factor (Mfidi 2017; Addy et al., 2021). The fear of confidentiality breaches among professionals further prevented adolescents from seeking help (Carlson et al., 2021; Khombo et al., 2023; Panford-Quainoo et al., 2024; Nkosi, 2025). Adolescents also encountered structural barriers, including geographical distance to services and financial constraints.

On the other hand, key themes involving facilitators of MH help-seeking included school-based MH programmes (Carlson et al., 2021), family and community support, peer networks (Addy et al., 2021; Khombo et al., 2023; Nkosi, 2025) and increased MHL through school-based education (Mfidi 2017; Meza et al., 2020; Panford-Quainoo et al., 2024; Mukuna, 2025). Supportive school environments with trained counsellors and MH education initiatives encourage help-seeking. Family and community support, particularly from parents, teachers and peers, plays a crucial role in promoting MH awareness and normalising help-seeking behaviours.

In a few instances, supportive school MH environments in this QES were linked to policies derived from national MH plans or school health legislation. However, where counselling services and psychoeducational interventions were described, most studies reflected implementation at institutional or externally supported levels rather than through formally operationalised, legally enforced policy mechanisms. This implies that MH policies are often present across SSA contexts but are typically weakly enacted, poorly resourced and fail to regularly adhere to international models (e.g., the World Health Organization Health Promoting Schools framework)(Meza et al., 2020; Freeman, 2022; Panford-Quainoo et al., 2024). Therefore, supportive school environments would seem to develop organically *via* grassroots/local mandates or external intervention, as opposed to operating as structured systems.

However, these facilitators did not operate independently. Improved MHL appeared to strengthen the impact of peer support and school-based services, as adolescents who recognised symptoms were more likely to utilise trusted peers and available professionals. Similarly, supportive school climates amplified the effects of confidentiality assurance, suggesting that structural and relational elements jointly shape help-seeking behaviour.

Although presented as distinct themes for analytical purposes, several of these themes overlap bidirectionally. The facilitators and barriers were not mutually exclusive and affected help-seeking behaviour both positively and negatively. For example, family and parental authority functioned as barriers when imposed through discouragement or negativity towards help-seeking but as facilitators when built on trust and open communication. Similarly, peer relationships, school climate and religious influence changed from risk factors to protective factors based on experiences. These results show that identical structural and sociocultural factors can facilitate or hinder adolescent use of MH care.

Compared to previous reviews conducted in other geographic regions, our findings corroborate previous evidence in low- and middle-income countries (LMICs), where stigma and a lack of knowledge about MH are significant problems (Aguirre Velasco et al., 2020; Barrow and Thomas, 2022; van den et al., 2023). The studies included in this review reaffirm that perceived stigma, fear of discrimination and low awareness of MH services deter adolescents from seeking formal help (Mfidi, 2017; Meza et al., 2020; Carlson et al., 2021; Abdulsalam et al., 2023). Evidence from qualitative reviews of school-based interventions in high-income settings shows that targeted support often triggers anticipated stigma and peer judgement (Gronholm et al., 2018). This aligns with the evidence in this synthesis, where fear of labelling and visibility influenced adolescents’ reluctance to seek support.

Similar patterns of stigma and discrimination have been observed in LMICs in Southeast Asia and Latin America in countries such as Haiti, Honduras and Nicaragua (González Moller et al., 2021; Javed et al., 2021; Lien et al., 2024), where traditional beliefs and misinformation about MHPs compounded these barriers (Saade et al., 2023). Unlike high-income settings, where MH education and awareness campaigns have played a significant role in promoting help-seeking behaviour (Newlove-Delgado et al., 2021; Adams et al., 2022; Westberg et al., 2022; Habgood et al., 2024), SSA continues to face challenges in implementing school-based MH interventions because of limited resources, a lack of trained professionals and inadequate policy frameworks (Addy et al., 2021; Brits, 2021; Aboagye et al., 2022).

Furthermore, while prior research underscores the significance of implementing professional MH services (Abdulsalam et al., 2023; Adams et al., 2022; Breslin et al., 2022), this QES reveals that adolescents in SSA frequently depend more on informal support systems, such as teachers and religious leaders, rather than on formal MH professionals (Carlson et al., 2021; Panford-Quainoo et al., 2024; Mushonga et al., 2025). This reliance on informal networks is evident in studies such as Carlson et al. (2021) and Khombo et al. (2023), in which adolescents reported seeking guidance from family members or school staff rather than engaging with trained professionals. Stigma, low MHL and structural access barriers in high-income contexts have been described elsewhere as barriers to MH help-seeking (Jörns-Presentati et al., 2021; Hlophe et al., 2023; van den et al., 2023).

However, these barriers operate differently in SSA settings, where help-seeking tends to rely more on family authority figures, religious beliefs and informal or community support systems (Addy et al., 2021; Panford-Quainoo et al., 2024). MH symptoms in SSA settings are more likely to be attributed to spiritual causes, personal weakness or family dishonour and driven by concealment behaviour (Mayston et al., 2020; Asiimwe et al., 2023; Hlongwane and Juby, 2023). In higher-income settings, findings on adolescents’ help-seeking have more commonly pointed to individual-level attitudinal barriers or perceptions of dissatisfaction with services (Gulliver et al., 2010; Radez et al., 2021), rather than contextualised explanations of distress shaped by culture or community. Findings from SSA expand upon previous theoretical models of help-seeking globally by highlighting the embeddedness of help-seeking behaviour within adolescents’ families, cultural backgrounds and religious practices (Rickwood and Thomas, 2012). This signals a need for global MH theory to expand upon individualistic, cognition-based models of help-seeking behaviour to better integrate structural and sociocultural barriers.

This finding contrasts with findings from high-income countries, where adolescents are more likely to access formal psychological support within schools or community-based services (Rickwood and Thomas, 2012; Heerde and Hemphill, 2018; Lauzier-Jobin and Houle, 2022). The preference for informal networks suggests that interventions in SSA should leverage these existing support structures while simultaneously strengthening access to professional services through policy changes and infrastructure development.

### Strengths and limitations

There are a number of notable strengths associated with this QES. First, we implemented a systematic and rigorous search strategy, and multiple reviewers independently conducted both the study selection and data extraction while complying with predefined inclusion and exclusion criteria. The study performed a critical appraisal and thematic synthesis according to established methodologies, which maintained methodological rigour.

Despite its strengths, the breadth of perspectives captured in the findings remains limited, as only eight qualitative studies met the inclusion requirements. Research shows a high concentration of studies in South Africa and West Africa, which may limit the generalisability of findings to other SSA regions. Most of the findings depended on barriers reported by individuals, which might obscure true institutional and systemic limitations. In addition, our review only included studies published in English, which again may limit generalisability. Future studies should focus on conducting qualitative research in regions that lack representation while examining structural and policy-level influences on adolescent MH service use.

### Implications for future research and policy

The findings of this QES have critical implications for research, policy and practice. From a research perspective, future research should include longitudinal studies designed to track how adolescent help-seeking behaviours evolve over time (Eigenhuis et al., 2021; Abdulsalam et al., 2023). Research should further investigate the sustained effects of current MH interventions (van den Broek et al., 2023; Carlson et al., 2021) while evaluating new methods that aim to improve adolescent engagement with MH services (Barrow and Thomas, 2022; Mabrouk et al., 2022). Research urgently requires randomised controlled trials (RCTs) to examine specific factors impacting help-seeking behaviour, especially in settings that remain unexplored (O’Dea et al., 2020; Radez et al., 2021; O’Neill et al., 2023).

Policymakers must prioritise the integration of MHL into school curricula within the broader education system (Addy et al., 2021; Carlson et al., 2021; Khombo et al., 2023). This approach requires the creation and dissemination of educational materials suitable for adolescents to learn about identifying MHPs and developing stress management skills while understanding how to access support systems (Mushonga et al., 2025; Nkosi, 2025). School-based MH services, like counselling, must be implemented so adolescents can obtain confidential treatment in a familiar setting (Azfredrick, 2015; Carlson et al., 2021). This proposed service model holds significant potential for rural and underserved areas due to the current shortage of specialised care options. Achieving this goal requires essential investment in school counsellor training programmes and infrastructure development (Azfredrick, 2015; Panford-Quainoo et al., 2024).

From a practice standpoint, school MH professionals must focus on building trust and maintaining confidentiality in their practice with adolescents (Carlson et al., 2021; Yao et al., 2021). Establishing clear communication about privacy and consent can alleviate the fear of seeking help for MHPs (Yao et al., 2021; Haraldsson et al., 2022). Schools and communities should also collaborate to actively combat MH stigma through awareness campaigns that aim to normalise seeking support (Chinawa et al., 2015; Bella-Awusah et al., 2019; Cefai et al., 2021). A multi-sectoral approach involving healthcare providers, educators and policymakers is essential for creating a holistic, adolescent-centred support system that addresses the full spectrum of MH needs (van den Broek et al., 2023; Iswanto and Ayubi, 2023; Lien et al., 2024).

## Conclusion

Overall, our findings revealed that schools offer a powerful yet often overlooked platform for the cost-effective adolescent MH services. The synthesis further provides insight into adolescents’ experiences and barriers to develop foundational guidance for context-sensitive and inclusive school MH programmes. The data demonstrate a pressing requirement for qualitative research which takes context into account to direct policy and practice while transforming schools into accessible hubs for MHL, early intervention and normalisation of help-seeking for MHPs.

In addition to institutional reforms, school-based MH programmes in SSA should also be mindful of the social context in which adolescents reside. Aspects that may influence whether adolescents seek help include parental hierarchy and authority, community attitudes about sharing problems and spiritual beliefs about mental illness. Behavioural interventions that do not consider these relational factors may be ineffective if adolescents are unable or unwilling to access help. Culturally appropriate engagement efforts at school and community levels, such as family sensitisation and engaging religious/community leaders, could build goodwill towards school-based MH programmes.

Notably, the QES reveals significant gaps in our knowledge of adolescent help-seeking behaviours within school environments in SSA. Quantitative studies dominate the field, limiting our understanding of adolescents lived experiences and help-seeking behaviours within school settings. While some subgroups, such as pregnant adolescents or those with substance use issues, have received focused attention, the broader spectrum of adolescent voices in SSA remains underexplored. Future research should further integrate qualitative methods, which are sensitive to context and accurately capture the range of school environments, to examine MH help-seeking in SSA adolescents. Strengthening school-based MH systems in SSA, therefore, represents not only a response to unmet need, but also a strategic investment in the long-term social, educational and psychological well-being of future generations.

## Supporting information

10.1017/gmh.2026.10250.sm001 Kakinda et al. supplementary material Kakinda et al. supplementary material

## Data Availability

Data sharing does not apply to this article as no new data were created or analysed.

## Supplementary material

The supplementary material for this article can be found at http://doi.org/10.1017/gmh.2026.10250.

## Long descriptions

### Table 1. Long description

The table consists of two columns: Element and Details.

* Sample S: School-going adolescents aged 10 to 19 years residing in Sub-Saharan Africa.

* Phenomenon of interest P I: Perceived barriers and facilitators influencing formal and informal mental health help-seeking behaviours among school-going adolescents in S S A.

* Design D: Qualitative studies including focus groups, in-depth interviews, group discussions, and paired interviews; mixed-method studies to extract qualitative data.

* Evaluation E: Perceived barriers, facilitators, views, experiences, attitudes, challenges, hindrances, access and referral processes.

* Research type R: Peer-reviewed qualitative studies focusing on mental health help-seeking behaviours in S S A.

* Keywords: A comprehensive list of search terms including Help-seeking behaviour, Mental health help-seeking, Utilization of mental health services, Attitude towards seeking help, Access to mental health services, Help-seeking intention, Attitude towards counselling, Attitude towards psychotherapy, Africa or African help-seeking behaviour, Sub-Saharan or Sub-Sahara or Africa south of the Sahara help-seeking behaviour, Africa or African mental health, Sub-Saharan or Sub-Sahara or Africa south of the Sahara help-seeking attitude, Africa or African help-seeking attitude, Africa or African help-seeking intention, Sub-Saharan or Sub-Sahara or Africa south of the Sahara help-seeking intention, Barrier, Challenge, Obstacle, Impediment, Hindrance, Obstruction, Hurdle, Delay, Access, and Refer.

Navigate back to Table 1..

### Table 2. Long description

The table consists of three columns titled Criteria, Inclusion, and Exclusion, with six rows of data.

* Geographic scope. Inclusion: Studies conducted in Sub-Saharan Africa. Exclusion: Studies conducted outside Sub-Saharan Africa.

* Phenomenon of interest. Inclusion: Studies focusing on perceived barriers and facilitators to formal and informal mental health help-seeking behaviours. Exclusion: Studies not examining perceived barriers or facilitators to mental health help-seeking.

* Population. Inclusion: school-going adolescents aged 10 to 19 years. Exclusion: Studies not involving school-going adolescents aged 10 to 19.

* Study design. Inclusion: Studies utilise qualitative research methods such as interviews, focus groups, qualitative content analysis or ethnographic observations. Exclusion: Quantitative-only studies, studies that did not employ qualitative methods.

* Publication type. Inclusion: Peer-reviewed full-text articles published in English. Exclusion: Grey literature, non-peer-reviewed sources, non-English publications.

* Time frame. Inclusion: Published from 1990 onwards. Exclusion: Published prior to 1990.

Navigate back to Table 2..

### Figure 1. Long description

The flowchart is divided into three vertical phases: Identification, Screening, and Included.

1. Identification Phase:

- Top-left box: Records identified from Databases n equals 1,905. Breakdown includes Cinahl n equals 125, PubMed n equals 1,054, E R I C n equals 105, Psyc I N F O n equals 112, S C O P U S n equals 509, and Google Scholar n equals 51.

- Top-right box: Records removed before screening. Duplicate records removed n equals 298, records marked as ineligible by automation tools n equals 267, and records removed for other reasons n equals 31.

2. Screening Phase:

- Second box down on left: Records screened n equals 1,309. An arrow points right to Records excluded during title and abstract screening n equals 1,197.

- Third box down on left: Reports sought for retrieval n equals 112. An arrow points right to Reports not retrieved n equals 10.

- Fourth box down on left: Reports assessed for eligibility n equals 102. An arrow points right to a detailed list of Reports excluded totaling 90. Reasons include: Not conducted in Sub-Saharan Africa n equals 29, Population not school-going adolescents 10 to 19 years n equals 19, Quantitative design studies n equals 17, Full text not accessible n equals 01, No focus on perceived barriers or facilitators to mental health help-seeking n equals 13, and Not peer-reviewed n equals 11.

3. Included Phase:

- Bottom-left box: Final studies included in review n equals 12.

Navigate back to Figure 1..

### Table 3. Long description

The table consists of 8 columns: Title, References, Country/study setting, Participants n; Type, Study design, Data collection, Data analysis, and Contribution to review question.

Key entries include:

* Mfidi 2017: South Africa, 15 participants (teachers and nurses). Highlights institutional barriers like limited training and unclear referral pathways.

* Abdulsalam et al. 2023: Nigeria, 24 students. Identifies stigma and gendered expectations as barriers; peer support as a facilitator.

* van de Water et al. 2018: South Africa, 10 adolescents. Focuses on how stigma regarding P T S D interventions discourages engagement.

* Coetzee et al. 2022: South Africa, 66 stakeholders. Discusses confidentiality and parental involvement in psychoeducational programs.

* Meza et al. 2020: Kenya, 36 participants. Notes logistical constraints and the benefits of task-shifting.

* Carlson et al. 2021: Uganda, 59 participants. Emphasizes trust and institutional readiness.

* Khombo et al. 2023: Zimbabwe, 15 students. Links mental health literacy to service uptake.

* Addy et al. 2021: Ghana, 53 participants. Shows reliance on informal support systems like family and peers.

* Panford-Quainoo et al. 2024: Ghana, 15 counsellors. Highlights systemic challenges like role conflict and resource deficits.

* Mukuna 2025 and Nkosi 2025: South Africa, 8 learners each. Both focus on rural high school settings, literacy, and peer influence.

* Mushonga et al. 2025: Zimbabwe and Ghana, various stakeholders. Examines how parental engagement facilitates or constrains access to services.

Navigate back to Table 3..

### Table 4. Long description

The table consists of four columns and six numbered rows.

Row 1. Barrier Theme: Perceived stigma. Subthemes: Social or public stigma and cultural stigma. Number of Studies: 12.

Row 2. Barrier Theme: Gender norms as a barrier to help-seeking. Subthemes: Masculinity norms and stigma around emotional expression; gendered socialisation and structural constraints on help-seeking. Number of Studies: 9.

Row 3. Barrier Theme: Mental health literacy (Knowledge, misconceptions and awareness gaps). Subthemes: Poor recognition of M H P s; limited knowledge and misbeliefs about self-help and professional support options; negative attitudes hindering M H P recognition and help-seeking; limited knowledge of how to access mental health information. Number of Studies: 11.

Row 4. Barrier Theme: Privacy, trust and confidentiality concerns with M H professionals. Subthemes: Fear of confidentiality breaches; inadequate counselling environments and lack of privacy; distrust in mental health professionals. Number of Studies: 7.

Row 5. Barrier Theme: Lack of accessibility and availability of M H services. Subthemes: Distance to services; lack of mental health professionals; inadequate resources. Number of Studies: 9.

Row 6. Barrier Theme: Family and parental attitudes, peer influence and alternative support. Subthemes: Family control and restriction of help-seeking; preference for informal and relational support networks; cultural and normative beliefs shaping help-seeking. Number of Studies: 7.

Navigate back to Table 4..

### Table 5. Long description

The table consists of four columns: an index number, Facilitator theme, Sub-themes, and Number of studies.

* Row 1: Mental health education and literacy enhancement. Sub-themes include promotion of mental health literacy, access to information and resources, integration into the school curriculum, and parental mental health education. Supported by 12 studies.

* Row 2: Supportive school environment or climate. Sub-themes include school mental health initiatives, school mental health policies, and institutional commitment. Supported by 10 studies.

* Row 3: Improved professional services. Sub-themes include availability of trained and specialized mental health professionals, and confidential, gender-sensitive, and culturally appropriate care. Supported by 8 studies.

* Row 4: Family, community involvement and peer support. Sub-themes include family communication and emotional support, peer support and shared coping, and community-based support and outreach. Supported by 8 studies.

* Row 5: Improved service accessibility and affordability. Sub-themes include policy and financial investment in mental health services, and school-based integration of mental health services. Supported by 6 studies.

Navigate back to Table 5..

## References

[r1] Abdulsalam I, Ahmed Tharbe IH and Mohd Jaladin RA (2023) Help-seeking: A qualitative study of help-seeking behaviours of students in public secondary schools in Northeast Nigeria. Pertanika Journal of Social Sciences and Humanities 31(3), 1297–1316. 10.47836/pjssh.31.3.19.

[r2] Aboagye RG, Ahinkorah BO, Seidu A-A, Okyere J, Frimpong JB and Kumar M (2022) In-school adolescents’ loneliness, social support, and suicidal ideation in sub-Saharan Africa: Leveraging global school health data to advance mental health focus in the region. PLoS One 17(11), e0275660. 10.1371/journal.pone.0275660. 36350793 PMC9645589

[r3] Adams C, Gringart E and Strobel N (2022) Explaining adults’ mental health help-seeking through the lens of the theory of planned behavior: A scoping review. Systematic Reviews 11(1), 160. 10.1186/s13643-022-02034-y. 35945633 PMC9361557

[r4] Addy ND, Agbozo F, Runge-Ranzinger S and Grys P (2021) Mental health difficulties, coping mechanisms and support systems among school-going adolescents in Ghana: A mixed-methods study. PLoS One 16(4), e0250424. 10.1371/journal.pone.0250424. 33886671 PMC8062044

[r5] Aguirre Velasco A, Cruz ISS, Billings J, Jimenez M and Rowe S (2020) What are the barriers, facilitators and interventions targeting help-seeking behaviours for common mental health problems in adolescents? A systematic review. BMC Psychiatry 20(1), 293. 10.1186/s12888-020-02659-0. 32527236 PMC7291482

[r6] Akın A and Sarrar L (2024) Understanding adolescent mental health in the COVID-19 era: A psychodynamic approach. Children 11(7), 772. 10.3390/children11070772. 39062222 PMC11274799

[r7] Akl EA, Khabsa J, Iannizzi C, Piechotta V, Kahale LA, Barker JM, McKenzie JE, Page MJ, Skoetz N and PRISMA-LSR Group (2024) Extension of the PRISMA 2020 statement for living systematic reviews (PRISMA-LSR): checklist and explanation 387, e079183. 10.1136/bmj-2024-079183.

[r8] Al Omari O, Khalaf A, Al Sabei S, Al Hashmi I, Al Qadire M, Joseph M and Damra J (2022) Facilitators and barriers of mental health help-seeking behaviours among adolescents in Oman: A cross-sectional study. Nordic Journal of Psychiatry 76(8), 591–601. 10.1080/08039488.2022.2038666. 35209780

[r9] Al-Shannaq Y and Aldalaykeh M (2023) Suicide literacy, suicide stigma, and psychological help seeking attitudes among Arab youth. Current Psychology 42(8), 6532–6544. 10.1007/s12144-021-02007-9. 34177209 PMC8214717

[r10] Amone-P’Olak K, Kakinda AI, Kibedi H and Omech B (2023) Barriers to treatment and care for depression among the youth in Uganda: The role of mental health literacy. Frontiers in Public Health 11. 10.3389/fpubh.2023.1054918. PMC10029729 36960365

[r11] Aromataris E and Riitano D (2014) Systematic reviews. AJN The American Journal of Nursing 114(5), 49–56. 10.1097/01.NAJ.0000446779.99522.f6. 24759479

[r12] Asiimwe R, Racheal K, Dwanyen L and Kasujja R (2023) Sociocultural considerations of mental health care and help-seeking in Uganda. SSM - Mental Health 4, 100232. 10.1016/j.ssmmh.2023.100232.

[r13] Azfredrick EC (2015) Use of counselling services by school-attending adolescent girls in Nigeria. Journal of Child and Adolescent Mental Health 27(1), 1–10. 10.2989/17280583.2014.953955. 25531745

[r14] Babatunde GB, van Rensburg AJ, Bhana A and Petersen I (2021) Barriers and facilitators to child and adolescent mental health services in low-and-middle-income countries: A scoping review. Global Social Welfare 8(1), 29–46. 10.1007/s40609-019-00158-z.

[r15] Bach NX, Bich NN and Anh DH (2023) Stigma, help-seeking and factors associated with mental health literacy in adults: A literature review. VNU Journal of Science: Medical and Pharmaceutical Sciences 39(4). 10.25073/2588-1132/vnumps.4561.

[r16] Barrow E and Thomas G (2022) Exploring perceived barriers and facilitators to mental health help-seeking in adolescents: A systematic literature review. Educational Psychology in Practice 38(2), 173–193. 10.1080/02667363.2022.2051441.

[r17] Batten J and Brackett A (2022) Ensuring rigor in systematic reviews: Part 6, reporting guidelines. Heart & Lung 52, 22–25. 10.1016/j.hrtlng.2021.11.002. 34823052

[r18] Bella-Awusah T, Ani C, Ajuwon A and Omigbodun O (2019) Should mental health be addressed in schools? Preliminary views of in-school adolescents in Ibadan, Nigeria. International Journal of School Health 6(2), e85937. 10.5812/intjsh.85937.

[r19] Birrell L, Grummitt L, Smout S, Maulik P, Teesson M and Newton N (2025) Debate: Where to next for universal school-based mental health interventions? Child and Adolescent Mental Health 30(1), 92–95. 10.1111/camh.12749. 39789719 PMC11754696

[r20] Bramer W and Bain P (2017) Updating search strategies for systematic reviews using EndNote. Journal of the Medical Library Association: JMLA 105(3), 285–289. 10.5195/jmla.2017.183. 28670219 PMC5490709

[r21] Bramer WM, de Jonge GB, Rethlefsen ML, Mast F and Kleijnen J (2018) A systematic approach to searching: An efficient and complete method to develop literature searches. Journal of the Medical Library Association: JMLA 106(4), 531–541. 10.5195/jmla.2018.283. 30271302 PMC6148622

[r22] Bramer WM, Rethlefsen ML, Kleijnen J and Franco OH (2017) Optimal database combinations for literature searches in systematic reviews: A prospective exploratory study. Systematic Reviews 6(1), 245. 10.1186/s13643-017-0644-y. 29208034 PMC5718002

[r23] Braun V and Clarke V (2023) Toward good practice in thematic analysis: Avoiding common problems and be(com)ing aknowingresearcher. International Journal of Transgender Health 24(1), 1–6. 10.1080/26895269.2022.2129597. 36713144 PMC9879167

[r301] Breslin G, Shannon S, Prentice G, Rosato M and Leavey G (2022) Adolescent mental health help-seeking from family and doctors: Applying the theory of planned behaviour to the Northern Ireland schools and wellbeing study. 28(4), 522–535.. Child Care in Practice 28(4), 522–535.

[r24] Brits E (2021) High mental health burden for Africa’s youth. Nature Africa. 10.1038/d44148-021-00097-y.

[r25] Brouwers EPM (2020) Social stigma is an underestimated contributing factor to unemployment in people with mental illness or mental health issues: Position paper and future directions. BMC Psychology 8(1), 36. 10.1186/s40359-020-00399-0. 32317023 PMC7171845

[r26] Campbell OLK, Bann D and Patalay P (2021) The gender gap in adolescent mental health: A cross-national investigation of 566,829 adolescents across 73 countries. SSM - Population Health 13, 100742. 10.1016/j.ssmph.2021.100742. 33748389 PMC7960541

[r27] Carlson C, Namy S, Nakuti J, Mufson L, Ikenberg C, Musoni O, Hopson L, Anton-Erxleben K, Naker D and Wainberg ML (2021) Student, teacher, and caregiver perceptions on implementing mental health interventions in Ugandan schools. Implementation Research and Practice 2, 26334895211051290. 10.1177/26334895211051290. PMC9762807 36540326

[r28] Carmona C, Baxter S and Carroll C (2022) The conduct and reporting of qualitative evidence syntheses in health and social care guidelines: A content analysis. BMC Medical Research Methodology 22(1), 267. 10.1186/s12874-022-01743-1. 36224536 PMC9554851

[r29] Cefai C, Simões C and Caravita SCS (2021) A Systemic, Whole-School Approach to Mental Health and Well-Being in Schools in the EU (Report). European Union. Retrieved from https://www.um.edu.mt/library/oar/handle/123456789/101739

[r30] Chinawa JM, Manyike PC, Obu HA, Aronu AE, Odutola O and Chinawa AT (2015) Depression among adolescents attending secondary schools in south East Nigeria. Annals of African Medicine 14(1), 46–51. 10.4103/1596-3519.148737. 25567695

[r31] Clough BA, Nazareth SM, Day JJ and Casey LM (2024) A comparison of mental health literacy, attitudes, and help-seeking intentions among domestic and international tertiary students. In Migration and Wellbeing. London: Routledge. 10.4324/9781032633503-11.

[r32] Codjoe L, Barber S, Ahuja S, Thornicroft G, Henderson C, Lempp H and N’Danga-Koroma J (2021) Evidence for interventions to promote mental health and reduce stigma in Black faith communities: Systematic review. Social Psychiatry and Psychiatric Epidemiology 56(6), 895–911. 10.1007/s00127-021-02068-y. 33866378 PMC8053235

[r33] Coetzee BJ, Gericke H, Human S, Stallard P and Loades M (2022) What should a universal school-based psychoeducational programme to support psychological well-being amongst children and young people in South Africa focus on and how should it be delivered? A multi-stakeholder perspective. School Mental Health 14(1), 189–200. 10.1007/s12310-021-09465-3. 35273653 PMC8897361

[r34] Cooke A, Smith D and Booth A (2012) Beyond PICO. Qualitative Health Research 22(10), 1435–1443. 10.1177/1049732312452938. 22829486

[r35] Cosma A, Black M, Vuckovic S, Pavic I, Fonseca H and Lazzerini M (2025) The changing epidemiology of child and adolescent mental health requires an immediate policy response. Public Health in Practice 10, 100655. 10.1016/j.puhip.2025.100655. 41113959 PMC12528862

[r36] Duong MT, Bruns EJ, Lee K, Cox S, Coifman J, Mayworm A and Lyon AR (2021) Rates of mental health service utilization by children and adolescents in schools and other common service settings: A systematic review and meta-analysis. Administration and Policy in Mental Health and Mental Health Services Research 48(3), 420–439. 10.1007/s10488-020-01080-9. 32940884

[r37] Eigenhuis E, Waumans RC, Muntingh ADT, Westerman MJ, van Meijel M, Batelaan NM and van Balkom AJLM (2021) Facilitating factors and barriers in help-seeking behaviour in adolescents and young adults with depressive symptoms: A qualitative study. PLoS One 16(3), e0247516. 10.1371/journal.pone.0247516. 33684154 PMC7939362

[r38] Flemming K and Noyes J (2021) Qualitative evidence synthesis: Where are we at? International Journal of Qualitative Methods 20, 1609406921993276. 10.1177/1609406921993276.

[r39] Freeman M (2022) The world mental health report: Transforming mental health for all. World Psychiatry 21(3), 391–392. 10.1002/wps.21018. 36073688 PMC9453907

[r40] Fulbright H and Evans C (2024) Finding full texts in bulk: A comparison of EndNote 20 versus Zotero 6 using the University of York’s subscriptions. Journal of the Medical Library Association 112(3), 214–224. 10.5195/jmla.2024.1880. 39308912 PMC11412117

[r41] Gibbs KD, Loveless J and Crane S (2022) A guide to using technological applications to facilitate systematic reviews. Worldviews on Evidence-Based Nursing 19(6), 442–449. 10.1111/wvn.12611. 36380454 PMC11465921

[r42] González Moller J, Heaphy G and Urrutia Ortiz J (2021) The feasibility of systemic interventions for the prevention and treatment of children and adolescent mental health difficulties in Latin American countries: A mixed studies systematic review. Journal of Family Therapy 43(4), 576–620. 10.1111/1467-6427.12327.

[r43] Gronholm PC, Nye E and Michelson D (2018) Stigma related to targeted school-based mental health interventions: A systematic review of qualitative evidence. Journal of Affective Disorders 240, 17–26. 10.1016/j.jad.2018.07.023. 30041074

[r44] Gulliver A, Griffiths KM and Christensen H (2010) Perceived barriers and facilitators to mental health help-seeking in young people: A systematic review. BMC Psychiatry 10(1), 113. 10.1186/1471-244X-10-113. 21192795 PMC3022639

[r45] Habgood E, Gandhi S, Smith R, Hearps S, Hiscock H, Oberklaid F, Raniti M and Darling S (2024) Pilot evaluation of an innovative school-based mental health literacy program for teachers and students: The decode mental health and wellbeing program. The Australian Educational Researcher. 10.1007/s13384-024-00774-5.

[r46] Haddaway NR, Page MJ, Pritchard CC and McGuinness LA (2022) PRISMA2020: An R package and shiny app for producing PRISMA 2020-compliant flow diagrams, with interactivity for optimised digital transparency and open synthesis. Campbell Systematic Reviews 18(2), e1230. 10.1002/cl2.1230. 36911350 PMC8958186

[r47] Haile ZT (2022) Critical appraisal tools and reporting guidelines. Journal of Human Lactation 38(1), 21–27. 10.1177/08903344211058374. 34791933

[r48] Haraldsson J, Pingel R, Nordgren L, Johnsson L, Kristiansson P and Tindberg Y (2022) Confidentiality matters! Adolescent males’ views of primary care in relation to psychosocial health: A structural equation modelling approach. Scandinavian Journal of Primary Health Care 40(4), 438–449. 10.1080/02813432.2022.2144999. 36458627 PMC9848349

[r49] Hayes D, Mansfield R, Mason C, Santos J, Moore A, Boehnke J, Ashworth E, Moltrecht B, Humphrey N, Stallard P, Patalay P and Deighton J (2024) The impact of universal, school based, interventions on help seeking in children and young people: A systematic literature review. European Child & Adolescent Psychiatry 33(9), 2911–2928. 10.1007/s00787-022-02135-y. PMC9837763 36637482

[r50] Heerde JA and Hemphill SA (2018) Examination of associations between informal help-seeking behavior, social support, and adolescent psychosocial outcomes: A meta-analysis. Developmental Review 47, 44–62. 10.1016/j.dr.2017.10.001.

[r51] Hlongwane N and Juby V (2023) Knowledge, attitudes, and help-seeking behaviour for mental illness in a Christian community. South African Journal of Psychiatry, 29, 2139. 10.4102/sajpsychiatry.v29i0.2139. PMC10696576 38059199

[r52] Hlophe LD, Tamuzi JL, Shumba CS and Nyasulu PS (2023) Barriers and facilitators to anti-retroviral therapy adherence among adolescents aged 10 to 19 years living with HIV in sub-Saharan Africa: A mixed-methods systematic review and meta-analysis. PLoS One 18(5), e0276411. 10.1371/journal.pone.0276411. 37200399 PMC10194875

[r53] Iswanto ED and Ayubi D (2023) The relationship of mental health literacy to help-seeking behavior: Systematic review. Journal of Social Research 2(3), 755–764. 10.55324/josr.v2i3.726.

[r54] Ivey C and Crum J (2018) Choosing the right citation management tool: Endnote, Mendeley, Refworks, or Zotero. Journal of the Medical Library Association : JMLA 106(3), 399–403. 10.5195/jmla.2018.468.

[r55] Javed A, Lee C, Zakaria H, Buenaventura RD, Cetkovich-Bakmas M, Duailibi K, Ng B, Ramy H, Saha G, Arifeen S, Elorza PM, Ratnasingham P and Azeem MW (2021) Reducing the stigma of mental health disorders with a focus on low- and middle-income countries. Asian Journal of Psychiatry 58, 102601. 10.1016/j.ajp.2021.102601. 33611083

[r56] Jessiman P, Kidger J, Spencer L, Geijer-Simpson E, Kaluzeviciute G, Burn A, Leonard N and Limmer M (2022) School culture and student mental health: A qualitative study in UK secondary schools. BMC Public Health 22(1), 619. 10.1186/s12889-022-13034-x. 35351062 PMC8964383

[r57] Jorm AF, Korten AE, Jacomb PA, Christensen H, Rodgers B and Pollitt P (1997) ‘Mental health literacy’: A survey of the public’s ability to recognise mental disorders and their beliefs about the effectiveness of treatment. The Medical Journal of Australia 166(4), 182–186. 10.5694/j.1326-5377.1997.tb140071.x. 9066546

[r58] Jörns-Presentati A, Napp A-K, Dessauvagie AS, Stein DJ, Jonker D, Breet E, Charles W, Swart RL, Lahti M, Suliman S, Jansen R, van den HLL, Seedat S and Groen G (2021) The prevalence of mental health problems in sub-Saharan adolescents: A systematic review. PLoS One 16(5), e0251689. 10.1371/journal.pone.0251689. 33989357 PMC8121357

[r59] Khombo S, Khombo K, Stoddart RS, Sifelani I and Sibanda T (2023) Knowledge, attitudes, and uptake of mental health services by secondary school students in Gweru, Zimbabwe. Frontiers in Psychology 14. 10.3389/fpsyg.2023.1002948. PMC9930152 36818083

[r60] Kilpatrick K, Savard I, Audet L-A, Costanzo G, Khan M, Atallah R, Jabbour M, Zhou W, Wheeler K, Ladd E, Gray DC, Henderson C, Spies LA, McGrath H and Rogers M (2024) A global perspective of advanced practice nursing research: A review of systematic reviews. PLoS One 19(7), e0305008. 10.1371/journal.pone.0305008. 38954675 PMC11218965

[r61] Kip EC, Udedi M, Kulisewa K, Go VF and Gaynes BN (2022) Stigma and mental health challenges among adolescents living with HIV in selected adolescent-specific antiretroviral therapy clinics in Zomba District, Malawi. BMC Pediatrics 22(1), 253. 10.1186/s12887-022-03292-4. 35524228 PMC9077887

[r62] Lauzier-Jobin F and Houle J (2022) A comparison of formal and informal help in the context of mental health recovery. International Journal of Social Psychiatry 68(4), 729–737. 10.1177/00207640211004988. 33736520 PMC9014766

[r63] Lewin S, Booth A, Glenton C, Munthe-Kaas H, Rashidian A, Wainwright M, Bohren MA, Tunçalp Ö, Colvin CJ, Garside R, Carlsen B, Langlois EV and Noyes J (2018) Applying GRADE-CERQual to qualitative evidence synthesis findings: Introduction to the series. Implementation Science 13(S1), 2. 10.1186/s13012-017-0688-3. 29384079 PMC5791040

[r64] Lewin S, Glenton C, Munthe-Kaas H, Carlsen B, Colvin CJ, Gülmezoglu M, Noyes J, Booth A, Garside R and Rashidian A (2015) Using qualitative evidence in decision making for health and social interventions: An approach to assess confidence in findings from qualitative evidence syntheses (GRADE-CERQual). PLoS Medicine 12(10), e1001895. 10.1371/journal.pmed.1001895. 26506244 PMC4624425

[r65] Lien Y-J, Chen L, Cai J, Y-H W and Liu Y-Y (2024) The power of knowledge: How mental health literacy can overcome barriers to seeking help. American Journal of Orthopsychiatry 94(2), 127–147. 10.1037/ort0000708. 37917500

[r66] Long HA, French DP and Brooks JM (2020) Optimising the value of the critical appraisal skills programme (CASP) tool for quality appraisal in qualitative evidence synthesis. Research Methods in Medicine & Health Sciences 1(1), 31–42. 10.1177/2632084320947559.

[r67] Lu W, Todhunter-Reid A, Mitsdarffer ML, Muñoz-Laboy M, Yoon AS and Xu L (2021) Barriers and facilitators for mental health service use among racial/ethnic minority adolescents: A systematic review of literature. Frontiers in Public Health 9. 10.3389/fpubh.2021.641605. PMC7982679 33763401

[r68] Ma KKY, A-M B and Anderson JK (2023) Review: School-based mental health literacy interventions to promote help-seeking – A systematic review. Child and Adolescent Mental Health 28(3), 408–424. 10.1111/camh.12609. 36377083

[r69] Mabrouk A, Mbithi G, Chongwo E, Too E, Sarki A, Namuguzi M, Atukwatse J, Ssewanyana D and Abubakar A (2022) Mental health interventions for adolescents in sub-Saharan Africa: A scoping review. Frontiers in Psychiatry 13. 10.3389/fpsyt.2022.937723. PMC9429610 36061286

[r70] Maeda Y, Caskurlu S, Kozan K and Kenney RH (2023) Development of a critical appraisal tool for assessing the reporting quality of qualitative studies: A worked example. Quality & Quantity 57(2), 1011–1031. 10.1007/s11135-022-01403-y.

[r71] Mayston R, Frissa S, Tekola B, Hanlon C, Prince M and Fekadu A (2020) Explanatory models of depression in sub-Saharan Africa: Synthesis of qualitative evidence. Social Science & Medicine 246, 112760. 10.1016/j.socscimed.2019.112760. 32006814 PMC7014569

[r72] McPhail L, Thornicroft G and Gronholm PC (2024) Help-seeking processes related to targeted school-based mental health services: Systematic review. BMC Public Health 24(1), 1217. 10.1186/s12889-024-18714-4. 38698391 PMC11065683

[r73] Meza RD, Kiche S, Soi C, Khairuzzaman AN, Nales CJR, Whetten K, Wasonga AI, Amanya C and Dorsey S (2020) Barriers and facilitators of child and guardian attendance in task-shifted mental health services in schools in western Kenya. Global Mental Health 7, e16. 10.1017/gmh.2020.9. 32742674 PMC7379319

[r74] Mfidi FH (2017) Mental health issues of school-going adolescents in high schools in the eastern cape, South Africa. Africa Journal of Nursing and Midwifery 19(3), 1–13. 10.25159/2520-5293/2219.

[r75] Mindu T, Mutero IT, Ngcobo WB, Musesengwa R and Chimbari MJ (2023) Digital mental health interventions for young people in rural South Africa: Prospects and challenges for implementation. International Journal of Environmental Research and Public Health 20(2), 1453. 10.3390/ijerph20021453. 36674209 PMC9859354

[r76] Mukuna KR (2025) Exploring adolescent learners’ perceptions of mental health and behavioural needs in a rural school setting. International Journal of Studies in Sexuality Education 1(2), 11–18. 10.38140/ijsse.v1i2.2101.

[r77] Mushonga RH, Jopling R, Glozah F, Kamvura TT, Dodd S, Gudyanga D, Maramba A, Dambayi E, Ayuure CA, Bere T, Achana FS, Owusu L, Chibanda D, Abas M, Weobong B and Kumwenda M (2025) Parental involvement in school-based mental health interventions for young people in low-resource settings: A qualitative study from Zimbabwe and Ghana. PLoS One 20(5), e0322954. 10.1371/journal.pone.0322954. 40343947 PMC12063816

[r78] Mutahi J, Larsen A, Cuijpers P, Peterson SS, Unutzer J, McKay M, John-Stewart G, Jewell T, Kinuthia J, Gohar F, Lai J, Wamalwa D, Gachuno O and Kumar M (2022) Mental health problems and service gaps experienced by pregnant adolescents and young women in sub-Saharan Africa: A systematic review. EClinicalMedicine 44, 44. 10.1016/j.eclinm.2022.101289. PMC8851289 35198916

[r79] Newlove-Delgado T, McManus S, Sadler K, Thandi S, Vizard T, Cartwright C, Ford T and Mental Health of Children and Young People group (2021) Child mental health in England before and during the COVID-19 lockdown. The Lancet. Psychiatry 8(5), 353–354. 10.1016/S2215-0366(20)30570-8. 33444548 PMC8824303

[r80] Nguyen H, Conway M-L, Murphy D, Brady A and Hennessy E (2025) Predictors of help-seeking intention among young people: A common-sense model based study. Children and Youth Services Review 178, 108549. 10.1016/j.childyouth.2025.108549.

[r81] Nkosi L (2025) Adolescent learners’ attitudes towards mental and behavioural health needs at a rural high school. International Journal of Studies in Sexuality Education 1(2), 26–32. 10.38140/ijsse.v1i2.2103.

[r82] O’Dea B, Han J, Batterham PJ, Achilles MR, Calear AL, Werner-Seidler A, Parker B, Shand F and Christensen H (2020) A randomised controlled trial of a relationship-focussed mobile phone application for improving adolescents’ mental health. Journal of Child Psychology and Psychiatry, and Allied Disciplines 61(8), 899–913. 10.1111/jcpp.13294. 32683737 PMC7496128

[r83] O’Neill A, Stapley E, Rehman I and Humphrey N (2023) Adolescent help-seeking: An exploration of associations with perceived cause of emotional distress. Frontiers in Public Health 11, 1183092. 10.3389/fpubh.2023.1183092. 37849721 PMC10578439

[r84] Ojagbemi A and Gureje O (2021) Sociocultural contexts of mental illness experience among Africans. Transcultural Psychiatry 58(4), 455–459. 10.1177/13634615211029055. 34427458

[r85] Ouzzani M, Hammady H, Fedorowicz Z and Elmagarmid A (2016) Rayyan—A web and mobile app for systematic reviews. Systematic Reviews 5(1), 210. 10.1186/s13643-016-0384-4. 27919275 PMC5139140

[r86] Page MJ, McKenzie JE, Bossuyt PM, Boutron I, Hoffmann TC, Mulrow CD, Shamseer L, Tetzlaff JM, Akl EA, Brennan SE, Chou R, Glanville J, Grimshaw JM, Hróbjartsson A, Lalu MM, Li T, Loder EW, Mayo-Wilson E, McDonald S, McGuinness LA, Stewart LA, Thomas J, Tricco AC, Welch VA, Whiting P and Moher D (2021) The PRISMA 2020 statement: An updated guideline for reporting systematic reviews. BMJ 372. 10.1136/bmj.n71. PMC8005924 33782057

[r87] Panford-Quainoo E, Oppong Asante K and Osei-Tutu A (2024) Practices and challenges of counselling in selected senior high schools in Accra, Ghana. Journal of Psychologists and Counsellors in Schools 34(2), 183–196. 10.1177/20556365231224478.

[r88] Radez J, Reardon T, Creswell C, Lawrence PJ, Evdoka-Burton G and Waite P (2021) Why do children and adolescents (not) seek and access professional help for their mental health problems? A systematic review of quantitative and qualitative studies. European Child & Adolescent Psychiatry 30(2), 183–211. 10.1007/s00787-019-01469-4. 31965309 PMC7932953

[r89] Rickwood D, Deane F, Wilson C and Ciarrochi J (2005) Young people’s help-seeking for mental health problems. Australian E-Journal for the Advancement of Mental Health 4(3), 218–251. 10.5172/jamh.4.3.218.

[r90] Rickwood D and Thomas K (2012) Conceptual measurement framework for help-seeking for mental health problems. Psychology Research and Behavior Management 5, 173–183. 10.2147/PRBM.S38707. 23248576 PMC3520462

[r110] Saade S, Parent-Lamarche A, Khalaf T, Makke S and Legg A (2023) What barriers could impede access to mental health services for children and adolescents in Africa? A scoping review. BMC Health Services Research 23(1), 348. 37024835 10.1186/s12913-023-09294-x PMC10080850

[r91] Sarikhani Y, Bastani P, Rafiee M, Kavosi Z and Ravangard R (2021) Key barriers to the provision and utilization of mental health services in low-and middle-income countries: A scope study. Community Mental Health Journal 57(5), 836–852. 10.1007/s10597-020-00619-2. 32285371

[r92] Seedaket S, Turnbull N, Phajan T and Wanchai A (2020) Improving mental health literacy in adolescents: Systematic review of supporting intervention studies. Tropical Medicine & International Health: TM & IH 25(9), 1055–1064. 10.1111/tmi.13449. 32478983

[r93] Singh OP (2021) Comprehensive mental health action plan 2013–2030: We must rise to the challenge. Indian Journal of Psychiatry 63(5), 415–417. 10.4103/indianjpsychiatry.indianjpsychiatry_811_21. 34789927 PMC8522612

[r94] Singh JA, Siddiqi M, Parameshwar P and Chandra-Mouli V (2019) World Health Organization guidance on ethical considerations in planning and reviewing research studies on sexual and reproductive health in adolescents. The Journal of Adolescent Health 64(4), 427–429. 10.1016/j.jadohealth.2019.01.008. 30904091 PMC6496912

[r111] Thapa A, Cohen J, Guffey S and Higgins-D’Alessandro A (2013) A review of school climate research. Review of educational research 83(3), 357–385.

[r95] Tong A, Flemming K, McInnes E, Oliver S and Craig J (2012) Enhancing transparency in reporting the synthesis of qualitative research: ENTREQ. BMC Medical Research Methodology 12(1), 181. 10.1186/1471-2288-12-181. 23185978 PMC3552766

[r96] van de Water T, Rossouw J, van der Watt ASJ, Yadin E and Seedat S (2018) Adolescents’ experience of stigma when accessing school-based PTSD interventions. Qualitative Health Research 28(7), 1088–1098. 10.1177/1049732318761365. 29542399

[r97] van den Broek M, Gandhi Y, Sureshkumar DS, Prina M, Bhatia U, Patel V, Singla DR, Velleman R, Weiss HA, Garg A, Sequeira M, Pusdekar V, Jordans MJD and Nadkarni A (2023) Interventions to increase help-seeking for mental health care in low- and middle-income countries: A systematic review. PLOS Global Public Health 3(9), e0002302. 10.1371/journal.pgph.0002302. 37703225 PMC10499262

[r98] Wang M-T and Degol JL (2016) School climate: A review of the construct, measurement, and impact on student outcomes. Educational Psychology Review 28(2), 315–352. 10.1007/s10648-015-9319-1.

[r99] Westberg KH, Nyholm M, Nygren JM and Svedberg P (2022) Mental health problems among young people—A scoping review of help-seeking. International Journal of Environmental Research and Public Health 19(3), 1430. 10.3390/ijerph19031430. 35162452 PMC8835517

[r100] World Health Organization (2018) *Global Status Report on Alcohol and Health 2018.* Geneva: World Health Organization. Available at https://iris.who.int/handle/10665/274603 (accessed 8 January 2024).

[r101] World Health Organization (2022) World Mental Health Report: Transforming Mental Health for all. Geneva: World Health Organization. Available at https://www.who.int/publications/i/item/9789240049338

[r102] Yao E, Li Y, Wang C and Hui J (2021) Understanding confidentiality breach in adolescent mental health sessions: An integrated model of culture and parenting. Ethics & Behavior 31(4), 245–256. 10.1080/10508422.2020.1719105.

