# Supplementary material for: Barriers and facilitators of mental health help-seeking behaviours among school-going adolescents in Sub-Saharan Africa: A qualitative evidence synthesis
Source: Glob Ment Health (Camb). 2026 Jun 9;13:e133. doi: 10.1017/gmh.2026.10250 (PMC13373274; doi:10.1017/gmh.2026.10250)
Supplement: Kakinda et al. supplementary material [file S2054425126102507sup001.docx]

**Supplementary Materials for QES**

**Outline (Appendix)**

1. Supplementary Table 1: ENTREQ Guidelines Checklist (adapted from Tong et al.)
2. Supplementary Table 2: Study Evaluation Based on the CASP
3. Supplementary Table 3: Search strategy using the SPIDER tool
4. Supplementary Table 4: GRADE-CERQual Summary of Review Findings
5. Reflexivity Statement
6. Supplementary Table 5: Additional Quotations Supporting Themes and Subthemes
7. Supplementary Table 6: Search Strings for Each Database
8. Supplementary Table 7: MeSH (Medical Subject Headings) terms

**Appendix A**

**Supplementary Table 1**

ENTREQ Guidelines Checklist (adapted from Tong et al.)

Also included is a filled ENTREQ checklist showing how the synthesis met reporting expectations across all 21 items, from search processes and appraisal through to synthesis methods and transparency standards. Clear, practical, and ready-to-use.

| **ENTREQ Item** | **Guide and Description** | **Notes/Details from the Document** | **Reported on** |
| --- | --- | --- | --- |
| **1. Aim of the synthesis** | State the research question that the synthesis addresses. | The aim to understand barriers and facilitators to mental health help-seeking behaviours is stated. | Pg 03-04 |
| **2. Synthesis Methodology** | Describe the methodology used for the synthesis (e.g., QES) or theoretical framework that underpins the synthesis and describe the rationale for choice of methodology. | Qualitative evidence synthesis (QES) is identified as the chosen method. | Pg 04 |
| **3. Approach to Searching** | Describe how searches were conducted (e.g., SPIDER tool, PRISMA protocol). | The SPIDER tool was used, and the search strategy adhered to PRISMA guidelines. | Pg 06-07 |
| **4. Inclusion Criteria** | Specify the inclusion/exclusion criteria used to include studies (e.g., population, intervention, study design). | Criteria for study inclusion, such as age, region, and focus on mental health, are detailed. | Pg 07-08 |
| **5. Data sources** | List the databases or repositories searched. (e.g. electronic databases [MEDLINE, PsycINFO, CINAHL], Grey literature databases, relevant organisational websites, experts, experiential or social phenomenon- related terms, filters for qualitative research and search limits) | Databases included PubMed, PsycINFO, Scopus, CINAHL, ERIC, and Google Scholar. | Pg 06 |
| **6. Electronic search strategy** | Describe the literature search. Provide the specific search terms and combinations used. | Search terms and combinations for barriers and facilitators were described comprehensively. | Pg 06 |
| **7. Study screening methods** | Describe the process of screening and selecting studies. (e.g. title, abstracts and full text review and number of independent reviewers who screened studies) | Titles, abstracts, and full texts were screened in a structured process. | Pg 08-09 |
| **8.Study Characteristics** | Provide a summary of the characteristics of included studies. (e.g. year of publication, country, population, number of participants, data collection, methodology, analysis, and research questions) | Characteristics such as location, participants, and methods are summarized. | Pg 12–13 |
| **9.Study selection results** | Present the number of studies included and excluded at each stage. | Eight studies were included after screening 1,905 abstracts. | Pg 09-10 |
| **10. Rationale for appraisal** | Explain the reasons for assessing the quality of included studies. | Quality appraisal ensures methodological rigor and reliability of findings. | Pg 18 |
| **11. Appraisal of studies** | Report the criteria or tools used for quality appraisal. | The CASP checklist was used to appraise qualitative studies. | Pg 18 |
| **12. Appraisal process** | Describe how appraisal was conducted (e.g., number of reviewers). | Two reviewers independently appraised studies using the CASP checklist. | Pg 18 |
| **13. Appraisal Results** | Present the outcomes of the quality appraisal process. | All included studies met CASP criteria, with minor concerns noted for one study. | Pg 18 |
| **14. Data Extraction** | Describe how data were extracted from included studies. | Dual-review data extraction ensured consistency and reliability. | Pg 17 |
| **15. Software** | Specify software used for managing or analysing data, if any. | EndNote, Rayyan and NVivo was used for data management, screening and thematic analysis. | Pg 06-07 (EndNote, Rayyan) and pg 13–14 (NVivo) |
| **16. Number of reviewers** | State the number of reviewers involved in the synthesis process. Who participated in the coding and analysis. | Four reviewers participated, with two conducting screening and extraction. | Pg 08-09 |
| **17. Coding** | Explain the coding process used to analyse data. | Inductive thematic coding was conducted to identify patterns and themes. | Pg 13-14 |
| **18. Study comparison** | Describe how studies were compared to identify similarities or differences. | Studies were compared based on context (e.g., rural vs. urban) and participant demographics. | Pg 12-13 |
| **19. Derivation of themes** | Explain how themes were derived from the data. | Themes were derived inductively from quotes and patterns identified during coding. | Pg 13–14 |
| **20. Quotations** | Include illustrative quotations to support findings. | Participant quotes are used extensively to illustrate barriers and facilitators. | Pg 15 onwards |
| **21. Synthesis output** | Present rich, compelling, and useful results that go beyond a summary of the primary studies (e.g. new interpretation, models of evidence, conceptual models, analytical framework and development of a new theory or construct) | Themes and sub-themes are presented with implications for policy and practice. | Pg 14–20+ |

**Appendix B**

**Supplementary Table 2**

**Study Evaluation Based on the CASP**

**A completed Critical Appraisal Skills Programme (CASP) checklist summarising the appraisal for each study included in the qualitative evidence synthesis.**

| **Study / CASP Criteria** | **Abdulsalam et al., 2023** | **Van de Water et al., 2018** | **Coetzee et al., 2022** | **Meza et al., 2020** | **Carlson et al., 2021** | **Khombo et al., 2023** | **Mfidi, 2017** | **Addy et al., 2021** | **Panford-Quainoo et al., 2024** | **Mukuna, 2025** | **Nkosi, 2025** | **Mushonga et al., 2025** |
| --- | --- | --- | --- | --- | --- | --- | --- | --- | --- | --- | --- | --- |
| 1. Clear statement of aims | Yes | Yes | Yes | Yes | Yes | Yes | Yes | Yes | Yes | Yes | Yes | Yes |
| 2. Qualitative methodology appropriate | Yes | Yes | Yes | Yes | Yes | Yes | Yes | Yes | Yes | Yes | Yes | Yes |
| 3. Research design appropriate | Yes | Yes | Yes | Yes | Yes | Yes | Yes | Yes | Yes | Yes | Yes | Yes |
| 4. Recruitment strategy appropriate | Yes | Yes | Yes | Yes | Yes | Yes | Yes | Yes | Yes | Yes | Yes | Yes |
| 5. Data collection addressed issue | Yes | Yes | Yes | Yes | Yes | Yes | Yes | Yes | Yes | Yes | Yes | Yes |
| 6. Researcher–participant relationship considered | Yes | Yes | ID | ID | Yes | ID | Yes | Yes | ID | ID | ID | Yes |
| 7. Ethical issues considered | Yes | Yes | Yes | Yes | Yes | Yes | Yes | Yes | Yes | Yes | Yes | Yes |
| 8. Data analysis sufficiently rigorous | Yes | Yes | Yes | Yes | Yes | Yes | Yes | Yes | Yes | Yes | Yes | Yes |
| 9. Clear statement of findings | Yes | Yes | Yes | Yes | Yes | Yes | Yes | Yes | Yes | Yes | Yes | Yes |
| 10. Valuable research | Yes | Yes | Yes | Yes | Yes | Yes | Yes | Yes | Yes | Yes | Yes | Yes |

**Note. CASP = Critical Appraisal Skills Programme. ID = Inadequately Detailed.**

**Appendix C**

**Supplementary Table 3**

Search strategy using the SPIDER tool

| **Element** | **Search Terms** |
| --- | --- |
| **Sample** | "School-going adolescents" AND "Sub-Saharan Africa" |
| **Phenomenon of Interest** | "Help-seeking behaviour" OR "Mental health help-seeking" OR "Utilization of mental health services" OR "Attitude towards seeking help" |
| **Design** | "Qualitative research" OR "Focus groups" OR "In-depth interviews" OR "Group discussions" OR "Paired interviews" |
| **Evaluation** | "Perceived barriers" OR "Facilitators" OR "Views" OR "Experiences" OR "Attitudes" OR "Challenges" OR "Hindrances" OR "Access" OR "Referral processes" |
| **Research Type** | "Peer-reviewed" OR "Qualitative studies" OR "Focusing on mental health help-seeking behaviours in SSA" |

**Appendix D**

**Supplementary Table 4**

GRADE-CERQual Summary of Review Findings

| **Category** | **Review Finding (Theme)** | **Studies Contributing & Frequency** | **Methodological Limitations** | **Coherence** | **Adequacy of Data** | **Relevance** | **CERQual Assessment of Confidence in the Evidence** |
| --- | --- | --- | --- | --- | --- | --- | --- |
| Barriers | **Perceived stigma** (Social/public stigma; cultural stigma) | 12 | Minor concerns Minor – most studies were well-designed (per CASP) | High-consistent across contexts | High | High | High confidence |
|  | **Gender norms as barrier to help seeking** (Masculinity norms and stigma around emotional expression; gendered socialisation and structural constraints on help-seeking) | 9 | Moderate concerns- some studies lacked reflexivity on gender issues | Minor concerns | Moderate | High- relevant across cultural settings | Moderate confidence |
|  | **Mental health literacy (Knowledge, misconceptions, and awareness gaps)** (Poor recognition of MHPs; limited knowledge & misbeliefs about self-help and professional support options;  negative attitudes hindering MHP recognition and help-seeking; limited knowledge of how to access mental health information) | 11 | Moderate concerns | Moderate | High | High | High |
|  | **Privacy, trust, and confidentiality concerns with MH professionals** (Fear of confidentiality breaches; inadequate counselling environments and lack of privacy; distrust in mental health professionals) | 7 | Minor concerns | Minor concerns | Moderate | High | Moderate confidence |
|  | **Lack of accessibility and availability of MH services** (Distance to services; lack of mental health professionals; inadequate resources) | 9 | Moderate concerns | Moderate | High | High | High  confidence |
|  | **Family and parental attitudes, peer influence, and alternative support** (  Family control and restriction of help-seeking; preference for informal and relational support networks; cultural and normative beliefs shaping help-seeking  ) | 7 | Moderate concerns | Minor concerns | Moderate | Moderate | High |
| Facilitators | **Mental health education and literacy enhancement** (Promotion of mental health literacy; access to information and resources; integration into the school curriculum; parental mental health education) | 12 | Minor concerns | High | High | High | High confidence |
|  | **Supportive school environment or climate** (School mental health initiatives; school mental health policies and institutional commitment) | 10 | Moderate concerns | High | Moderate | High | Moderate confidence |
|  | **Improved professional services** (Availability of trained and specialised mental health professionals; confidential, gender-sensitive, and culturally appropriate care) | 8 | Moderate concerns | Minor concerns | Moderate | High | Moderate confidence |
|  | **Family, community involvement, and peer support** (Family communication and emotional support; peer support and shared coping; community-based support and outreach) | 8 | Moderate concerns | High | Moderate | Moderate | Moderate confidence |
|  | **Improved Services accessibility & affordability** (Policy and financial investment in mental health services; school-based integration of mental health services) | 6 | Moderate concerns | Moderate | Moderate | High | Moderate confidence |

**Reflexivity Statement**

To minimise bias and avoid dominance of a single perspective, the review involved a multidisciplinary team with diverse cultural and academic backgrounds. AIK, a counselling psychologist and mental health researcher based in the Global South, led the coding and appraisal process. He kept a reflexive diary to record his methodological choices and examine how his professional and personal experiences influenced his interpretations. Researchers RP and TC from the Global North brought their deep qualitative mental health research experience to support crucial synthesis decisions. KAP utilized his cross-cultural expertise and regional knowledge to steer context-specific interpretation. The team engaged in discussions where they evaluated assumptions and defined positionalities to enhance transparency. All reviewers committed to giving adolescents from low-resource areas a louder voice while they acknowledged these youths' perspectives contain valuable insights that mainstream mental health discussions typically overlook. The analytical focus and framing of findings were influenced by this approach.

**Appendix F**

**Supplementary Table 5**

Additional Quotations Supporting Themes and Subthemes

| **Superordinate (Analytic) Theme** | **Subordinate (Descriptive) Theme** | **Additional Quotations from Participants and Authors of Primary Studies** |
| --- | --- | --- |
| **1. Institutional and Resource Constraints** | Inaccessible or Overburdened Services | “We don't have enough counsellors for all of us. Sometimes you wait weeks before you get help.” (Participant, Mfidi, 2017)  *Schools in Eastern Cape lacked adequate personnel and infrastructure to provide sustained mental health support.* (Author, Mfidi, 2017) |
|  | Inconsistent Delivery of School-Based Services | “Sometimes the program runs, sometimes it stops. We never know when someone will come again.” (Participant, Meza et al., 2020)  *Task-shifted services faced logistical disruptions, limiting their effectiveness.* (Author, Meza et al., 2020) |
|  | Lack of Trained Mental Health Staff | “Teachers are not trained to help with mental problems. They focus on discipline.” (Participant, Coetzee et al., 2022)  *Stakeholders emphasised the need for training non-specialist staff to deliver effective interventions.* (Author, Coetzee et al., 2022) |
|  | Physical Space Constraints | “There is no private place to talk. Others can hear you.” (Participant, Carlson et al., 2021)  *Schools lacked appropriate physical infrastructure to deliver private or confidential services.* (Author, Carlson et al., 2021) |
| **2. Sociocultural Influences and Stigma** | Fear of Peer Judgement | “If others know you are going for counselling, they will laugh at you.” (Participant, van de Water et al., 2018)  *Stigma shaped adolescents’ willingness to attend PTSD interventions, often silencing help-seeking.* (Author, van de Water et al., 2018) |
|  | Misunderstanding Mental Health | “Mental illness is for crazy people. That’s what people say here.” (Participant, Khombo et al., 2023)  *Misconceptions remained high, contributing to low uptake of mental health services.* (Author, Khombo et al., 2023) |
|  | Gendered Perceptions of Vulnerability | “Boys are told to be strong. They don’t talk about feelings.” (Participant, Mfidi, 2017)  *Masculinity norms discouraged emotional expression among adolescent boys.* (Author, Mfidi, 2017) |
|  | Influence of Teachers and Parents | “My mother said I should focus on schoolwork, not emotions.” (Participant, Carlson et al., 2021)  *Caregiver and teacher attitudes directly influenced student participation in interventions.* (Author, Carlson et al., 2021) |
| **3. Adolescent Coping Strategies and Support Preferences** | Preference for Peer Support | “I talk to my friend. She understands and won’t tell anyone.” (Participant, Meza et al., 2020)  *Adolescents leaned on peers for emotional support when formal help felt risky or unavailable.* (Author, Meza et al., 2020) |
|  | Self-Reliance or Emotional Suppression | “You learn to deal with it. Crying won’t help.” (Participant, van de Water et al., 2018)  *Many participants adopted avoidant or internal coping as a survival mechanism.* (Author, van de Water et al., 2018) |
|  | Use of Faith and Cultural Resources | “We pray at home when someone is stressed.” (Participant, Khombo et al., 2023)  *Spiritual and cultural practices were part of informal coping systems.* (Author, Khombo et al., 2023) |
|  | Trust and Continuity in Care Relationships | “If you trust the person, you can go back again. If not, you stop.” (Participant, Carlson et al., 2021)  *Consistency and relational safety were key to sustained adolescent engagement in services.* (Author, Carlson et al., 2021) |

**Appendix G**

**Supplementary Table 6**

Search Strings for Each Database

| **Database** | **Search String** |
| --- | --- |
| **PubMed** | ((((((africa south of the sahara[MeSH Terms]) OR (Sub-Saharan africa[Title/Abstract])) OR (sub-sahara africa[Title/Abstract])) OR (ANGOLA[Title/Abstract] OR BENIN[Title/Abstract] OR BOTSWANA[Title/Abstract] OR "BURKINA FASO"[Title/Abstract] OR BURUNDI [Title/Abstract] OR "CAPE VERDE"[Title/Abstract] OR CAMEROON[Title/Abstract] OR "CENTRAL AFRICAN REPUBLIC"[Title/Abstract] OR CHAD[Title/Abstract] OR COMOROS[Title/Abstract] OR CONGO[Title/Abstract] OR "COTE D'IVOIRE"[Title/Abstract] OR "DEMOCRATIC REPUBLIC OF THE CONGO"[Title/Abstract] OR DJIBOUTI[Title/Abstract] OR "EQUATORIAL GUINEA"[Title/Abstract] OR ERITREA[Title/Abstract] OR ESWATINI[Title/Abstract] OR ETHIOPIA[Title/Abstract] OR GABON[Title/Abstract] OR GAMBIA[Title/Abstract] OR GHANA[Title/Abstract] OR GUINEA[Title/Abstract] OR GUINEA-BISSAU[Title/Abstract] OR KENYA[Title/Abstract] OR LESOTHO[Title/Abstract] OR LIBERIA[Title/Abstract] OR MALAWI[Title/Abstract] OR MALI[Title/Abstract] OR MADAGASCAR[Title/Abstract] OR MAURITANIA[Title/Abstract] OR MAURITIUS[Title/Abstract] OR MOZAMBIQUE[Title/Abstract] OR NAMIBIA[Title/Abstract] OR NIGER[Title/Abstract] OR NIGERIA[Title/Abstract] OR RWANDA[Title/Abstract] OR "SAO TOME AND PRINCIPE"[Title/Abstract] OR SENEGAL[Title/Abstract] OR "SIERRA LEONE"[Title/Abstract] OR SOMALIA[Title/Abstract] OR "SOUTH AFRICA"[Title/Abstract] OR "SOUTH SUDAN"[Title/Abstract] OR SUDAN[Title/Abstract] OR TANZANIA[Title/Abstract] OR TOGO[Title/Abstract] OR UGANDA[Title/Abstract] OR ZAMBIA[Title/Abstract] OR ZIMBABWE[Title/Abstract])) AND ((help-seeking[Title/Abstract]) OR Help seeking[Title/Abstract] OR “seek help”[Title/Abstract] OR health-seeking[Title/Abstract] OR ("Health Behaviour"[MeSH Terms] OR "Patient Acceptance of Health Care"[MeSH] OR “Help-seeking Behaviour”[MeSH Terms] OR care-seeking[Title/Abstract] OR reluctance[Title/Abstract] OR willingness[Title/Abstract] OR “Health Services Accessibility”[MeSH Terms] OR utilisation[Title/Abstract] OR utilization[Title/Abstract]))) AND (((Adolescent[MeSH Terms]) OR (adolescen*[Title/Abstract])) OR (teenage*[Title/Abstract] OR children[Title/Abstract] OR Youth[Title/Abstract] OR "Young people"[Title/Abstract]))) AND (((mental health[MeSH Terms]) OR (mental health[Title/Abstract])) OR (well-being[Title/Abstract] OR depression[Mesh] OR depression[Title/Abstract] OR psycho*[Title/Abstract] OR psychiatr*[Title/Abstract] OR anxiety[Title/Abstract] OR emotional[Title/Abstract])) |
| **Scopus** | (TITLE-ABS-KEY ("Sub-Saharan Africa" OR "sub-Sahara Africa" OR  Angola OR Benin OR Botswana OR "Burkina Faso" OR Burundi OR "Cape Verde" OR Cameroon OR  "Central African Republic" OR Chad OR Comoros OR Congo OR "cote d’Ivoire" OR  "Democratic Republic of the Congo" OR Djibouti OR "Equatorial Guinea" OR Eritrea OR  Eswatini OR Ethiopia OR Gabon OR Gambia OR Ghana OR guinea OR "Guinea-Bissau" OR  Kenya OR Lesotho OR Liberia OR Malawi OR Mali OR Madagascar OR Mauritania OR  Mauritius OR Mozambique OR Namibia OR Niger OR Nigeria OR Rwanda OR  "Sao tome and Principe" OR Senegal OR "Sierra Leone" OR Somalia OR  "South Africa" OR "South Sudan" OR Sudan OR Tanzania OR Togo OR Uganda OR  Zambia OR Zimbabwe))  AND  (TITLE-ABS-KEY ("help-seeking" OR help seeking OR "seek* help" OR health-seeking OR  "Health Behaviour" OR "Patient Acceptance of Health Care" OR  "Help-seeking Behaviour" OR care-seeking OR reluctance OR willingness OR  "Health Services Accessibility" OR utilisation OR utilization))  AND  (TITLE-ABS-KEY (adolescen* OR teenage OR children OR youth OR "young people"))  AND  (TITLE-ABS-KEY ("mental health" OR well-being OR depression OR psycho* OR psychiatr* OR  anxiety OR emotional)) |
| **PsycINFO** | ((((("Sub-Saharan Africa" OR "Africa South of the Sahara" OR ANGOLA OR BENIN OR BOTSWANA OR "BURKINA FASO" OR BURUNDI OR "CAPE VERDE" OR CAMEROON OR "CENTRAL AFRICAN REPUBLIC" OR CHAD OR COMOROS OR CONGO OR "COTE D'IVOIRE" OR "DEMOCRATIC REPUBLIC OF THE CONGO" OR DJIBOUTI OR "EQUATORIAL GUINEA" OR ERITREA OR ESWATINI OR ETHIOPIA OR GABON OR GAMBIA OR GHANA OR GUINEA OR GUINEA-BISSAU OR KENYA OR LESOTHO OR LIBERIA OR MALAWI OR MALI OR MADAGASCAR OR MAURITANIA OR MAURITIUS OR MOZAMBIQUE OR NAMIBIA OR NIGER OR NIGERIA OR RWANDA OR "SAO TOME AND PRINCIPE" OR SENEGAL OR "SIERRA LEONE" OR SOMALIA OR "SOUTH AFRICA" OR "SOUTH SUDAN" OR SUDAN OR TANZANIA OR TOGO OR UGANDA OR ZAMBIA OR ZIMBABWE)  AND  (help-seeking OR Help seeking OR "seek* help" OR health-seeking OR "Health Behaviour" OR "Patient Acceptance of Health Care" OR "Help-seeking Behaviour" OR care-seeking OR reluctance OR willingness OR "Health Services Accessibility" OR utilisation OR utilization)  AND  (adolescen* OR teenage* OR children OR youth OR "young people")  AND  (mental health OR well-being OR depression OR psycho* OR psychiatr* OR anxiety OR emotional))) |
| **CINAHL** | ((MH "Africa South of the Sahara" OR TI "Sub-Saharan Africa" OR AB "Sub-Saharan Africa" OR TI "sub-Sahara Africa" OR AB "sub-Sahara Africa"  OR TI (Angola OR Benin OR Botswana OR "Burkina Faso" OR Burundi OR "Cape Verde" OR Cameroon OR "Central African Republic" OR Chad OR Comoros OR Congo OR "Cote d'Ivoire" OR "Democratic Republic of the Congo" OR Djibouti OR "Equatorial Guinea" OR Eritrea OR Eswatini OR Ethiopia OR Gabon OR Gambia OR Ghana OR Guinea OR "Guinea-Bissau" OR Kenya OR Lesotho OR Liberia OR Malawi OR Mali OR Madagascar OR Mauritania OR Mauritius OR Mozambique OR Namibia OR Niger OR Nigeria OR Rwanda OR "Sao Tome and Principe" OR Senegal OR "Sierra Leone" OR Somalia OR "South Africa" OR "South Sudan" OR Sudan OR Tanzania OR Togo OR Uganda OR Zambia OR Zimbabwe)  OR AB (Angola OR Benin OR Botswana OR "Burkina Faso" OR Burundi OR "Cape Verde" OR Cameroon OR "Central African Republic" OR Chad OR Comoros OR Congo OR "Cote d'Ivoire" OR "Democratic Republic of the Congo" OR Djibouti OR "Equatorial Guinea" OR Eritrea OR Eswatini OR Ethiopia OR Gabon OR Gambia OR Ghana OR Guinea OR "Guinea-Bissau" OR Kenya OR Lesotho OR Liberia OR Malawi OR Mali OR Madagascar OR Mauritania OR Mauritius OR Mozambique OR Namibia OR Niger OR Nigeria OR Rwanda OR "Sao Tome and Principe" OR Senegal OR "Sierra Leone" OR Somalia OR "South Africa" OR "South Sudan" OR Sudan OR Tanzania OR Togo OR Uganda OR Zambia OR Zimbabwe))  AND   (MH "Help Seeking Behavior" OR TI help-seeking OR AB help-seeking OR TI Help seeking OR AB Help seeking OR TI "seek* help" OR AB "seek* help" OR TI health-seeking OR AB health-seeking OR MH "Health Behavior" OR MH "Patient Acceptance of Health Care" OR MH "Health Services Accessibility" OR TI care-seeking OR AB care-seeking OR TI reluctance OR AB reluctance OR TI willingness OR AB willingness OR TI utilization OR AB utilisation OR TI utilization OR AB utilization)   AND   (MH "Adolescents" OR TI adolescen* OR AB adolescen* OR TI teenage* OR AB teenage* OR TI children OR AB children OR TI youth OR AB youth OR TI "young people" OR AB "young people")   AND   (MH "Mental Health" OR TI "mental health" OR AB "mental health" OR TI well-being OR AB well-being OR MH "Depression" OR TI depression OR AB depression OR TI psycho* OR AB psycho* OR TI psychiatr* OR AB psychiatr* OR TI anxiety OR AB anxiety OR TI emotional OR AB emotional) |
| **ERIC** | (("Sub-Saharan Africa" OR "sub-Sahara Africa" OR ANGOLA OR BENIN OR BOTSWANA OR "BURKINA FASO" OR BURUNDI OR "CAPE VERDE" OR CAMEROON OR "CENTRAL AFRICAN REPUBLIC" OR CHAD OR COMOROS OR CONGO OR "COTE D'IVOIRE" OR "DEMOCRATIC REPUBLIC OF THE CONGO" OR DJIBOUTI OR "EQUATORIAL GUINEA" OR ERITREA OR ESWATINI OR ETHIOPIA OR GABON OR GAMBIA OR GHANA OR GUINEA OR GUINEA-BISSAU OR KENYA OR LESOTHO OR LIBERIA OR MALAWI OR MALI OR MADAGASCAR OR MAURITANIA OR MAURITIUS OR MOZAMBIQUE OR NAMIBIA OR NIGER OR NIGERIA OR RWANDA OR "SAO TOME AND PRINCIPE" OR SENEGAL OR "SIERRA LEONE" OR SOMALIA OR "SOUTH AFRICA" OR "SOUTH SUDAN" OR SUDAN OR TANZANIA OR TOGO OR UGANDA OR ZAMBIA OR ZIMBABWE) AND (help-seeking OR help seeking OR "seek* help" OR health-seeking OR care-seeking OR reluctance OR willingness OR utilisation OR utilization) AND (adolescen* OR teenage* OR children OR youth OR "young people") AND ("mental health" OR well-being OR depression OR psycho* OR psychiatr* OR anxiety OR emotional)) |

**Appendix H**

Supplementary Table 7: MeSH (Medical Subject Headings) terms

| **MeSH Term** | **Description / Focus** |
| --- | --- |
| Africa South of the Sahara | Geographical region (Sub-Saharan Africa) |
| Health Behaviour | Actions taken by individuals that affect health |
| Patient Acceptance of Health Care | Willingness of individuals to seek or receive care |
| Help-Seeking Behaviour | Actions aimed at obtaining assistance or help for problems |
| Health Services Accessibility | Availability and ease of access to healthcare services |
| Adolescent | Individuals aged 10–19 years |
| Mental Health | Emotional, psychological, and social well-being |
| Depression | Mood disorder involving persistent sadness |
