# Supplementary material for: Barriers and facilitators of mental health help-seeking behaviours among school-going adolescents in Sub-Saharan Africa: A qualitative evidence synthesis
Source: Glob Ment Health (Camb). 2026 Jun 9;13:e133. doi: 10.1017/gmh.2026.10250 (PMC13373274; doi:10.1017/gmh.2026.10250)
Supplement: Peer review history

# Reviewer report: Review: Barriers and facilitators of mental health help-seeking behaviours among school-going adolescents in Sub-Saharan Africa: A qualitative evidence synthesis — R0/PR2

**DOI:** 10.1017/gmh.2026.10250.pr2

Peer Review Report

For: Cambridge Prisms: Global Mental Health

Manuscript ID: GMH-2026-0007

Title: Promotion of Mental Health Help-Seeking Among School-Going Adolescents in Sub-Saharan Africa: A Qualitative Evidence Synthesis

Overall Recommendation: Accept with Minor Revisions

Section-by-Section Feedback for Authors

1. Abstract & Impact Statement: The abstract clearly summarizes the objective, methods, results, and implications. The Impact Statement effectively highlights the relevance of the findings for intervention design in SSA. However, there is some overlap in content between the Abstract and Impact Statement. To improve, refine the Impact Statement to focus more on the novel implications and translational value of the synthesis, rather than restating results. Also, ensure consistency in terminology (e.g., “MPHs” should be “MHPs”).

2. Introduction: This section provides a compelling rationale, clearly establishes the research gap, and situates the study within global and regional contexts. The objectives are well-defined. Authors should consider briefly noting how the SSA-focused review contributes to global understandings of adolescent help-seeking, particularly in terms of contextualizing universal barriers within region-specific realities.

3. Methods: The methodological approach of this manuscript is rigorous, transparent, and well-documented. The appropriate application of established frameworks, SPIDER, ENTREQ, PRISMA, CASP, and GRADE-CERQual, significantly enhances the study’s credibility. The search strategy is comprehensively described and appears fully reproducible. The “Data Extraction and Study Characteristics” section clearly outlines a systematic process, detailing the extraction of study characteristics, methodological features, participant details, and relevant constructs. However, the actual data representing these extracted elements, particularly the content spanning pages 13–17 intended as Table 3, is not presented in a coherent format. Currently, this content is fragmented, misaligned, and contains interrupted text, which prevents verification of the extraction process and systematic assessment of the included studies. To resolve this, the detailed study characteristics should be consolidated into a clear, standalone table, either in the main text or an appendix, with standardized domains such as title, author, year, country, sample, methods, and key findings. Furthermore, the reporting of study characteristics should be enhanced to include an in-depth analysis of how each included article addresses the specific research questions, moving beyond mere description to a more synthetic and critical presentation of the evidence, as this is a qualitative manuscript. This restructuring will ensure the methodological transparency described in the text is fully reflected in the presented data.

4. Results (Thematic Synthesis): The thematic presentation is logical, well-structured, and richly illustrated with participant quotes. The identification of six barriers and five facilitators is insightful and grounded in the data. Ensure terminological consistency across sections (e.g., “Mental Health Knowledge, misconceptions and awareness gaps” in Results vs. “low mental health literacy” in Discussion). Additionally, authors should consider deepening the analysis of facilitators by exploring interactions between themes (e.g., how mental health literacy and peer support jointly overcome stigma).

5. Discussion: The section effectively interprets findings, links them to existing literature, and draws meaningful implications for practice, policy, and research. The contrast between SSA and high-income settings is well-articulated. Authors are advised to include a dedicated paragraph that explicitly summarizes how SSA-specific findings (e.g., reliance on informal/religious networks, cultural stigma) compare and contrast with the global evidence base, and what unique lessons SSA offers to global mental health. Furthermore, authors should clarify any thematic overlap noted in the summary of barriers (e.g., “Mental Health Knowledge” vs. “low mental health literacy”).

6. Conclusion: This section succinctly reiterates the importance of schools as platforms for mental health support and calls for more context-sensitive qualitative research. However, the ending feels somewhat abrupt. Consider adding a final, impactful sentence that underscores the transformative potential of school-based mental health initiatives in SSA.

# Reviewer report: Review: Barriers and facilitators of mental health help-seeking behaviours among school-going adolescents in Sub-Saharan Africa: A qualitative evidence synthesis — R0/PR3

**DOI:** 10.1017/gmh.2026.10250.pr3

**Authors:** Zenah Nantumbwe

**Affiliations:** Psychiatry and Psychotherapy, University of Leipzig Faculty of Medicine, Germany

The manuscript addresses an important qualitative research gap in a global context specifically in Africa. The study design and methods are acceptable, and the results provide clear existing qualitative findings on the barriers and facilitators of both formal and informal mental health help-seeking behaviours among adolescents in Sub Saharan Africa.

I appreciate the clarity of the standard qualitative evidence synthesis (QES) for the methodology, and logical structure of the results presented.

A few minor revisions are suggested to the authors as below.

Abstract: Page 1 line 10, Clarify in the abstract which short form or abbreviation it is for mental health problems MPHs or MHPs.

Results: Pg 18-21, Considering ‘stigma is the most robust barrier,’ authors could consider clarifying the stigma variable and subthemes. For example, In Table 4 of barriers to mental health help seeking, ‘Social stigma, anticipated stigma, public stigma, cultural stigma, self-stigma are mentioned although some are not fully expounded or discussed. Consider if appropriate to merge subthemes like public stigma and social stigma as quotation used for public stigma, is also social stigma including friends and family(mothers) or reflect on using another clear distinguishing quotation.

Discussion: Pg. 42, In the discussion introduction it may be helpful to readers for authors to provide a global outlook or snapshot and then narrowing down to current work in Sub Saharan Africa rather than stating the research gap. Authors may consider how this synthesis fits into the continent or globally.

Implications or conclusion: Pg 45-47, Authors could consider providing some more social cultural perspective on SSA in the implications or conclusions.

Overall, this is a promising paper that would benefit from minor revision before publication.

# Reviewer report: Review: Barriers and facilitators of mental health help-seeking behaviours among school-going adolescents in Sub-Saharan Africa: A qualitative evidence synthesis — R0/PR4

**DOI:** 10.1017/gmh.2026.10250.pr4

**Authors:** Frederick Murunga Wekesah

**Affiliations:** Utrecht University

Overall

The review aimed to synthesise evidence on barriers and facilitators of both formal and informal mental health help-seeking behaviours among school-going adolescents. Some key structural and sociocultural inhibiting and facilitating factors were identified, against which key recommendations have been made.

1. This review would be incomplete without acknowledging the policy landscape that shapes healthcare in these settings, particularly the significant gaps in policies and guidelines on mental health for the general population, and even more so for school-going adolescents and young people.

For example, the reviewers found out that supportive school environments were facilitators for mental health help-seeking behaviours. But were these school environments anchored in policy or law? Did these schools implement the WHO Health Promoting Schools framework? ?

2. Because there is an overlap between barriers and facilitators i.e. same structural and sociocultural factors can be barriers and facilitators depending on how they present, perhaps the authors should present these side by side (under the same heading), showing how, say, family can be a barrier if it is dysfunctional, and if it offers support, it can be a facilitator. This way, one gets the full picture.

Methods

The methods as described are clear and straightforward. The five databases explored are relevant to the topic.

1. However, considering the authors were interested in qualitative evidence, it would have been great for them to review other niche-specific and region-specific journals that could publish research from the social sciences. These journals could be archived in AJOL, EMBASE, etc.

2. It is also notable that “the first search was conducted in March 2024, with updates in July 2024, and June 2025.” This is atypically long to carry out searches and updates of articles to include in a systematic review. It would be great to know why the authors felt this approach was optimal for this manuscript.

3. Why was “Manual citation tracking of pertinent articles conducted” in this day and age, when we have robust tools, some even freely available/open source, that they could use for this process?

4. It would be good to hear from the authors about how many instances they had to write to the selected authors of studies that were eligible, but where their academic library subscriptions did not allow them to access the full-text versions of papers. How successful was this approach?

5. Eligibility criteria: Why did the authors find it necessary to cite Zhang et al. 2023 when describing the inclusion of 10 – 19-year-olds in the study? Why do they feel this criterion is attributable to Zhang et al. 2023?

6. When discussing exclusion criteria, the authors should not wander outside the scope of sub-Saharan Africa. This sentence, “Studies outside Sub-Saharan Africa (SSA).....and quantitative studies were excluded from the synthesis”, is redundant. The authors should tell us why otherwise eligible studies in scope and type of design were excluded, and for what reason they were excluded.

7. Page 7”: this explanation “Endnote.....reference management software designed to organise the retrieved references” is superfluous for the audience the authors are producing this manuscript for.

8. Page 8, lines 44 – 45: This is the first time the authors indicate they also searched Google Scholar in addition to the five databases earlier listed.

9. Page 10: PRISMA Flowchart: Can the authors use the updated 2020 PRISMA Flowchart and Statement?

Findings

1. Theme 2: “Gender differences” seems to be an analysis of how the barriers and facilitators are distributed, as opposed to being a barrier or facilitator. The authors need to arrange the themes based on a framework and logical flow. Or else the authors need to rename “Gender” as a risk factor/barrier to mental health help-seeking among school-going adolescents.

2. The use of terms “one participant” and “one respondent” makes it sound as if the data are transcripts, and not findings from published manuscripts. The authors need to find better ways to report findings from the articles they cite.

3. The authors conflate ‘education’ with ‘knowledge/literacy’ across the manuscript. These terms must be properly reviewed and correctly presented.

Conclusions

This sentence, “Overall, our findings revealed that schools offer a powerful yet often overlooked platform for the economic delivery of adolescent mental health services” is not clear. Do the authors mean that schools offer an opportunity to deliver cost-effective mental health services to school-going adolescents?

# Editor report: Recommendation: Barriers and facilitators of mental health help-seeking behaviours among school-going adolescents in Sub-Saharan Africa: A qualitative evidence synthesis — R0/PR5

**DOI:** 10.1017/gmh.2026.10250.pr5

**Affiliations:** School of Public Health and Preventive Medicine, Monash University Faculty of Medicine Nursing and Health Sciences, Australia

Please address all the revisions suggested by the reviewers.

# Editor report: Decision: Barriers and facilitators of mental health help-seeking behaviours among school-going adolescents in Sub-Saharan Africa: A qualitative evidence synthesis — R0/PR6

**DOI:** 10.1017/gmh.2026.10250.pr6

**Affiliations:** London School of Hygiene & Tropical Medicine, United Kingdom of Great Britain and Northern Ireland

No accompanying comment.

# Reviewer report: Review: Barriers and facilitators of mental health help-seeking behaviours among school-going adolescents in Sub-Saharan Africa: A qualitative evidence synthesis — R1/PR8

**DOI:** 10.1017/gmh.2026.10250.pr8

**Authors:** Zenah Nantumbwe

**Affiliations:** Psychiatry and Psychotherapy, University of Leipzig Faculty of Medicine, Germany

The authors have adequately addressed comments with clear reponses and made changes to improve the manuscrpit.

# Reviewer report: Review: Barriers and facilitators of mental health help-seeking behaviours among school-going adolescents in Sub-Saharan Africa: A qualitative evidence synthesis — R1/PR9

**DOI:** 10.1017/gmh.2026.10250.pr9

At this point of the manuscript, I have no comments for the authors.

# Editor report: Recommendation: Barriers and facilitators of mental health help-seeking behaviours among school-going adolescents in Sub-Saharan Africa: A qualitative evidence synthesis — R1/PR10

**DOI:** 10.1017/gmh.2026.10250.pr10

**Affiliations:** School of Public Health and Preventive Medicine, Monash University Faculty of Medicine Nursing and Health Sciences, Australia

Thank you for submitting the revised manuscript. The reviewers agree that the revised paper is clear. However, before accepting the paper please make a final essential revision. 

The Introduction should end with an aim and a research question. It should not include a statement of what was done. Please remove the statement and place it in the Discussion and add a study aim.

Please make these final changes and submit your revised paper.

# Editor report: Decision: Barriers and facilitators of mental health help-seeking behaviours among school-going adolescents in Sub-Saharan Africa: A qualitative evidence synthesis — R1/PR11

**DOI:** 10.1017/gmh.2026.10250.pr11

**Affiliations:** London School of Hygiene & Tropical Medicine, United Kingdom of Great Britain and Northern Ireland

No accompanying comment.

# Editor report: Recommendation: Barriers and facilitators of mental health help-seeking behaviours among school-going adolescents in Sub-Saharan Africa: A qualitative evidence synthesis — R2/PR13

**DOI:** 10.1017/gmh.2026.10250.pr13

**Affiliations:** School of Public Health and Preventive Medicine, Monash University Faculty of Medicine Nursing and Health Sciences, Australia

Thank you for your response, I note that you retained the Impact Statement in the Introduction, please remove it and place it in the Discussion. I cannot reach a decision about publication until this change has been made.

# Editor report: Decision: Barriers and facilitators of mental health help-seeking behaviours among school-going adolescents in Sub-Saharan Africa: A qualitative evidence synthesis — R2/PR14

**DOI:** 10.1017/gmh.2026.10250.pr14

**Affiliations:** London School of Hygiene & Tropical Medicine, United Kingdom of Great Britain and Northern Ireland

No accompanying comment.

# Editor report: Recommendation: Barriers and facilitators of mental health help-seeking behaviours among school-going adolescents in Sub-Saharan Africa: A qualitative evidence synthesis — R3/PR16

**DOI:** 10.1017/gmh.2026.10250.pr16

**Affiliations:** School of Public Health and Preventive Medicine, Monash University Faculty of Medicine Nursing and Health Sciences, Australia

Thank you for submitting the revised paper, which I am pleased to accept.

# Editor report: Decision: Barriers and facilitators of mental health help-seeking behaviours among school-going adolescents in Sub-Saharan Africa: A qualitative evidence synthesis — R3/PR17

**DOI:** 10.1017/gmh.2026.10250.pr17

**Affiliations:** London School of Hygiene & Tropical Medicine, United Kingdom of Great Britain and Northern Ireland

No accompanying comment.
